# The Critical Role of YAP/BMP/ID1 Axis on Simulated Microgravity‐Induced Neural Tube Defects in Human Brain Organoids

**DOI:** 10.1002/advs.202410188

**Published:** 2024-12-10

**Authors:** Di Guo, Bin Yao, Wen‐Wei Shao, Jia‐Chen Zuo, Zhe‐Han Chang, Jian‐Xin Shi, Nan Hu, Shuang‐Qing Bao, Meng‐Meng Chen, Xiu Fan, Xiao‐Hong Li

**Affiliations:** ^1^ Academy of Medical Engineering and Translational Medicine Tianjin University Tianjin 300072 China; ^2^ State Key Laboratory of Advanced Medical Materials and Devices Tianjin 300072 China; ^3^ Haihe Laboratory of Brain‐Computer Interaction and Human‐Machine Integration Tianjin 300072 China

**Keywords:** adherens junctions, brain organoids, neural stem and progenitor cells, neural tube defects, simulated microgravity

## Abstract

Integrated biochemical and biophysical signals regulate embryonic development. Correct neural tube formation is critical for the development of central nervous system. However, the role of microgravity in neurodevelopment and its underlying molecular mechanisms remain unclear. In this study, the effects of stimulated microgravity (SMG) on the development of human brain organoids are investigated. SMG impairs N‐cadherin‐based adherens junction formation, leading to neural tube defects associated with dysregulated self‐renewal capacity and neuroepithelial disorganization in human brain organoids. Bulk gene expression analyses reveal that SMG alters Hippo and BMP signaling in brain organoids. The neuropathological deficits in SMG‐treated organoids can be rescued by regulating YAP/BMP/ID1 axis. Furthermore, sing‐cell RNA sequencing data show that SMG results in perturbations in the number and function of neural stem and progenitor cell subpopulations. One of these subpopulations senses SMG cues and transmits BMP signals to the subpopulation responsible for tube morphogenesis, ultimately affecting the proliferating cell population. Finally, SMG intervention leads to persistent neurologic damage even after returning to normal gravity conditions. Collectively, this study reveals molecular and cellular abnormalities associated with SMG during human brain development, providing opportunities for countermeasures to maintain normal neurodevelopment in space.

## Graphical abstract

This study reports the effects of simulated microgravity (SMG) on brain development using a human brain organoid model. SMG impairs the formation of adherens junctions, leading to neural tube defects associated with dysregulated self‐renewal capacity and neuroepithelial disorganization. SMG‐induced neuropathological defects can be rescued by modulating the YAP/BMP/ID1 axis, which provides a novel strategy to maintain normal neurodevelopment in space.

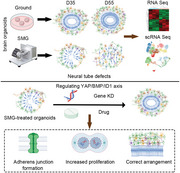

## 1 Introduction

Embryonic cells constantly receive mechanical stimuli generated by gravity, cell movement, cell‐extracellular matrix (ECM) interactions, and cell–cell interactions, during which myriad forces drive morphogenesis, representing the most underexplored frontier of developmental biology.^[^ ^1^, ^2^, ^3^ ^]^ The mechanical properties are thought to be critical for sculpting biological structures^[^ ^4^ ^]^ and controlling cell behavior, including cell migration,^[^ ^5^ ^]^ cell differentiation,^[^ ^6^, ^7^ ^]^ and neuronal growth.^[^ ^8^ ^]^ However, the exact effects of gravity on key stages of mammalian embryonic development remain enigmatic. As the development of life beyond earth has been a long‐standing human quest, numerous efforts have been devoted to studying the impact of embryo development in space microgravity.^[^ ^9^, ^10^, ^11^, ^12^, ^13^, ^14^ ^]^ Despite strong evidence that integrated biochemical and mechanical signals regulate embryonic development, the signaling pathways and cellular responses involved in space microgravity are still largely unknown.

The neural tube is regarded as the embryonic precursor of the central nervous system. Neural tube development depends on the ability of neural stem and progenitor cells (NSPCs) to divide to form correct diversity and arrangement of neuronal and glial cell progeny that perform the specialized functions in mature neural networks. The balance between self‐renewal and differentiation of NSPCs contributes to normal neurodevelopment.^[^ ^15^ ^]^ NSPCs have two points of adhesion responsible for neural tube morphogenesis. Their apical poles are attached to the ventricular luminal surface through N‐cadherin‐based adherens junctions (AJs), whereas their basal end‐feet adhere to the subpial ECM via integrin‐laminin interactions.^[^ ^16^ ^]^ Folding morphogenesis of neural tube requires apical contraction of neural ectoderm and basal adhesion mediated via ECM synthesis by non‐neural ectoderm.^[^ ^17^ ^]^ The inhibition of bone morphogenetic protein (BMP) signaling is required for growth and patterning of the neural tube.^[^ ^18^, ^19^ ^]^ During neural tube closure, BMP signaling from the epidermal ectoderm abolishes hinge point formation and induces neural tube defects (NTDs).^[^ ^18^, ^20^ ^]^ Smads 1, 5 and 9 in the BMP pathway are phosphorylated to accumulate in the nucleus and form transcriptional complexes that regulate hundreds of target genes. The inhibitor of DNA binding 1 (ID1), a target gene in BMP signaling, promotes the quiescence of NSPCs and repress their proliferation.^[^ ^21^, ^22^, ^23^ ^]^ With the rise of mechanobiology, there is increasing interest in understanding how mechanical stimuli affect neural tube development at the cellular level.^[^ ^24^, ^25^, ^26^ ^]^

Yes‐associated protein (YAP) is a mechanosensitive transcriptional activator that plays an important role in controlling the self‐renewal capacity and the number of NSPCs.^[^ ^27^, ^28^, ^29^, ^30^, ^31^ ^]^ Mechanical force triggers YAP nuclear entry to regulate target gene expression.^[^ ^32^, ^33^ ^]^ YAP cytoplasmic accumulation caused by NUAK family kinase 2 mutations impairs cytoskeletal processes that govern cell shape during neural tube closure.^[^ ^34^ ^]^ YAP activation by mechanical forces is required for notochord formation and neural tube patterning by inducing Forkhead box protein A2 and Shh expression,^[^ ^35^ ^]^ suggesting that YAP translates physical cues into molecular terms during neural tube morphogenesis. Previous studies have reported that YAP can integrate mechanical signals with BMP signaling in regulating AJs dynamics.^[^ ^36^ ^]^ However, the role of YAP on BMP signaling is controversial.^[^ ^36^, ^37^, ^38^ ^]^

Here, we used a human brain organoid model to examine in depth how SMG exposure affected neurodevelopment. Our data showed that SMG impeded the formation of N‐cadherin‐based AJs, resulting in NTDs associated with impaired proliferation and disorganization. The SMG‐induced defects could be reversed by regulating the YAP/BMP/ID1 axis. Additionally, we performed single‐cell transcriptomic analyses to uncover perturbations in NSPCs subpopulation and interactions under SMG conditions. A subpopulation of NSPCs may act as mechanosensor or gravireceptor, transmitting BMP signals to the subpopulation responsible for tube morphogenesis, which ultimately regulating the proliferating cell population. Finally, our study supported that SMG intervention led to persistent neurologic damage. Our findings may provide potential target genes and cell types for the prevention and treatment of SMG‐induced neurodevelopmental defects.

## 2 Results

### 2.1 Stimulated Microgravity Causes NTDs Associated with Impaired Proliferative Capacity and Structural Disorganization in Brain Organoids

**Figure 1:**
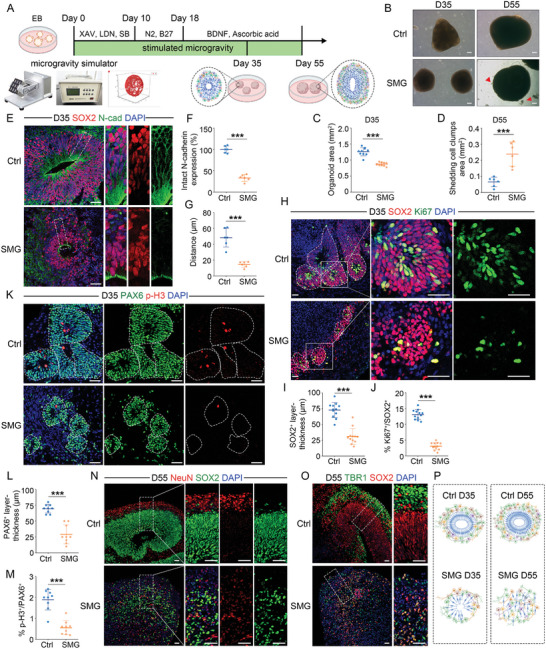
Stimulated microgravity causes NTDs associated with impaired proliferative capacity and structural disorganization in brain organoids. A) Schematic of experimental design. EB, embryoid bodies. B–D) Light images of control or SMG‐treated organoids on day 35 and day 55. Red arrows indicate the dropped cell clumps from organoids. Organoid area and cell mass shedding were measured on day 35 or 55, respectively. *n* = 10 individual organoids (C), *n* = 6 individual organoids (D). E–G) Immunostaining and quantification of the intact N‐cadherin expression and the distance from lumen border to first layer of nuclei at day 35. *n* = 6 individual organoids (F, G). H–M) Immunostaining and quantification of the thickness of SOX2^+^ and PAX6^+^ layer, and the cell proportion of Ki67^+^ and p‐H3^+^ proliferating cells in control and SMG‐treated organoids at day 35. White dashed lines indicate the borders of VZ‐like structures. *n* = 12 individual organoids (I, J), *n* = 9 individual organoids (L, M). N–P) Immunostaining and schematic of the organization of VZ‐ and CP‐like regions in control and SMG‐treated organoids. Created in BioRender (P). Scale bar, 200 µm (B), 50 µm (E, H, K, N, O). Values were mean ± SD. Statistical significance was determined by unpaired, two‐tailed Student's *t*‐test. ***p < 0.001.

To explore the effects of microgravity on human brain development, we generated brain organoids and loaded them onto a clinostat to stimulate microgravity. To understand the long‐term exposure of SMG on brain organoids, we started on day 0 when embryoid bodies were formed and examined on day 35 and 55 (**Figure** **1**A). We found that SMG significantly decreased the organoid sizes on day 35 (Figure 1B,C). On day 55, SMG deteriorated the growth of brain organoids, as evidenced by the shedding of cell clumps in the organoids (Figure 1B,D).

As the AJs play a crucial role in mechano‐sensing and epithelial morphogenesis,^[^ ^24^ ^]^ we detected the formation of N‐cadherin‐based AJs between neighboring NSPCs. Immunostaining and quantification of N‐cadherin expression revealed significant deficits in AJs formation by NSPCs in SMG‐treated organoids (Figure 1E,F). SMG‐treated organoids exhibited the characteristics of open NTDs, with a significantly reduced number of intact neural tube‐like structures (Figure S1A,B, Supporting Information). Moreover, SMG led to insufficient apical constriction, as assessed by the distance from lumen border to first layer of nuclei (Figure 1G). These results indicated that SMG impaired the AJs formation in brain organoids.

Considering that the AJs formation is essential for the self‐renewal of NSPCs and the polarized organization of brain,^[^ ^39^, ^40^, ^41^ ^]^ we co‐immunostained proliferative marker Ki67 or mitotic marker phospho‐histone 3 (p‐H3), with NSPCs marker SOX2 or PAX6 on day 35. We observed that SMG significantly reduced the proliferative capacity and maintenance of NSPCs in brain organoids (Figure 1H–M). A similar phenotype was observed in SMG‐treated brain organoids cultured in Matrigel‐containing medium (Figure S1C–G, Supporting Information). We also examined the effect of SMG on asymmetric and symmetric division of NSPC in brain organoids. The results showed that the effect of SMG on the division mode was not significant (Figure S1H,I, Supporting Information). To determine whether SMG influences the organization of brain organoids, we performed co‐immunostaining of cortical plate (CP)‐like layers (NeuN^+^ or TBR1^+^) and ventricular zone (VZ)‐like layers on day 35 and day 55. The neurons were extensively intermingled with NSPCs in SMG‐treated organoids, indicating that SMG induced aberrant neuronal positioning in the brain organoids (Figure 1N–P; Figure S1J, Supporting Information). Strikingly, we observed a lack of apparent ventricles in SMG‐treated organoids, whereas control organoids remained the large ventricle‐like structures at day 55 (Figure 1N,O). Collectively, SMG exposure impaired the AJs formation, leading to decreased NSPCs self‐renewal and maintenance as well as structural disorganization. These results provide important insights into how NSPCs respond to microgravity by interacting with themselves to regulate the size and cytoarchitecture of the nervous system.

### 2.2 SMG Leads to Altered Hippo and BMP Signaling in Brain Organoids

**Figure 2:**
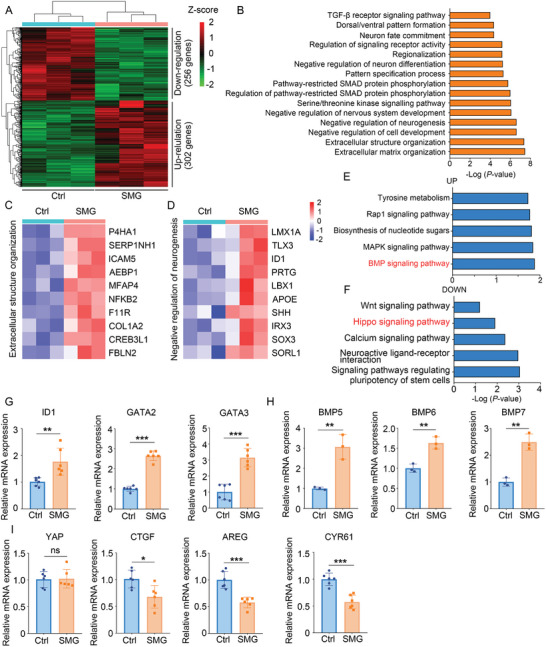
SMG leads to altered Hippo and BMP signaling in brain organoids. A) Heat maps of upregulated and downregulated differential expressed genes in SMG‐treated organoids on day 35. B) The 15 enriched GO terms of upregulated genes in SMG‐treated organoids versus control organoids at day 35. C,D) Normalized and scaled expression level of the top 10 significantly upregulated genes for each signature. E,F) Top 5 signaling pathways significantly increased (E) or decreased (F) in brain organoids under SMG conditions. G,H) The mRNA expression level of BMP target genes and BMPs in SMG‐treated organoids on day 35. *n* = 6 independent experiments (G), *n* = 3 independent experiments (H). I) The relative mRNA expression level of *YAP* gene and YAP target genes (*CTGF, AREG, CYR61*) in SMG‐treated organoids on day 35. *n* = 6 independent experiments. Values were mean ± SD. Statistical significance was determined by unpaired, two‐tailed Student's *t*‐test. ns: *P* > 0.05, *p < 0.05, **p < 0.01, ***p < 0.001.

To investigate the transcriptomic changes in response to SMG, we analyzed the transcriptional profiles of three batches of control and SMG‐treated organoids on day 35 by bulk RNA sequencing (RNA‐Seq). The heatmap of differential expressed genes showed that 256 genes were downregulated and 302 genes were upregulated in the SMG‐treated organoids compared with that in the control organoids (**Figure** **2**A). Gene Ontology (GO) analysis identified that the enrichment of extracellular structure organization, negative regulation of neurogenesis, pathway‐restricted SMAD protein phosphorylation, dorsal/ventral pattern formation in the SMG‐treated group compared to the control group (Figure 2B–D). Kyoto Encyclopedia of Genes and Genomes enrichment analyses showed that BMP signaling pathway was among the top upregulated transcriptional programs, while Hippo pathway target genes were among the top downregulated transcriptional programs (Figure 2E,F). We performed qRT‐PCR analysis to confirm these results. SMG‐treated organoids expressed significantly increased mRNA level of *ID1, GATA2, GATA3* (BMP target genes), *BMP5, BMP6, BMP7* (BMPs) than that in the control organoids (Figure 2G,H). Although SMG did not decrease the mRNA expression level of the Hippo pathway effector *YAP*, the target genes of YAP (*CTGF, AREG, CYR61*) were significantly decreased in SMG‐treated organoids (Figure 2I).

### 2.3 Inhibition of the BMP Signaling Significantly Ameliorates SMG‐Mediated Defects

**Figure 3:**
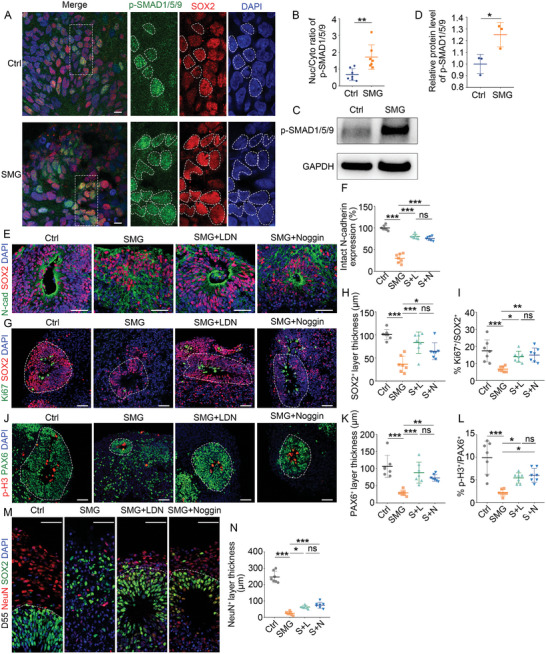
Inhibition of the BMP signaling significantly ameliorates SMG‐mediated defects. A,B) Fluorescence images and quantification of the ratio of phosphorylated SMAD 1/5/9 between nuclear and cytoplasmic intensity in NSPCs in control and SMG‐treated organoids on day 35. *n* = 7 individual organoids. C,D) Representative western blots of phosphorylated SMAD1/5/9 protein levels in control and SMG‐treated groups on day 35. *n* = 3 independent experiments. E,F) Immunostaining and quantification of the intact N‐cadherin expression in control (Ctrl), SMG‐treated (SMG), LDN193189‐treated under SMG conditions (SMG+LDN), and Noggin‐treated under SMG conditions (SMG+Noggin) organoids at day 35. *n* = 7 individual organoids. G–L) Immunostaining and quantification of the thickness of SOX2^+^ and PAX6^+^ layer, and the proportion of Ki67^+^ and p‐H3^+^ proliferating cells. *n* = 7 individual organoids. M,N) Immunostaining and quantification of the thickness of NeuN^+^ layer at day 55. *n* = 7 individual organoids. Scale bar, 10 µm (A), 50 µm (E, G, J, M). Values were mean ± SD. Statistical significance was determined by unpaired, two‐tailed Student's *t*‐test (B, D). Statistical significance was determined by one‐way ANOVA with the Dunnet post hoc test (F, H, I, K, L, N). ns: *P* > 0.05, *p < 0.05, **p < 0.01, ***p < 0.001.

The phosphorylated SMAD1/5/9 (p‐SMAD1/5/9) and the common mediator SMAD4 in the BMP signaling form heteromeric complexes, which then accumulate in the nucleus and regulate target gene transcription. As our RNA‐Seq analyses showed the enrichment of pathway‐restricted SMAD protein phosphorylation in SMG‐treated organoids, we examined the subcellular localization of p‐SMAD1/5/9 in response to SMG treatment. We found that there were more nuclear locations of p‐SMAD1/5/9 in the SMG‐treated organoids (**Figure** **3**A,B). Western blot analysis confirmed that p‐SMAD1/5/9 abundance significantly increased after SMG treatment (Figure 3C,D). These results suggested that SMG induced the activation of BMP signaling.

Since previous studies have revealed the existence of feedback loops between BMP signaling and N‐cadherin,^[^ ^42^ ^]^ we next asked whether inhibition of BMP signaling could ameliorate the deficits in AJs formation. We treated brain organoids with the BMP inhibitors LDN193189 and Noggin on day 18, and detected the growth of brain organoids on day 35 and day 55. Interestingly, the expression of N‐cadherin and the number of neural tube‐like structures were significantly increased in SMG‐treated organoids after LDN193189 and Noggin treatment on day 35 (Figure 3E,F; Figure S2A,B, Supporting Information). Furthermore, we examined the changes in proliferation capacity and structural organization after the use of BMP inhibitors on day 35 and day 55. We found that treatment with LDN193189 and Noggin partially rescued the reduction of NSPCs proliferation and maintenance in SMG‐treated organoids (Figure 3G–L; Figure S2C–F, Supporting Information). The decreased and ectopic neurogenesis in SMG‐treated organoids was ameliorated by LDN193189 and Noggin treatment (S2G–S2I). Since brain organoids contained extensive neurons at day 55, we also examined neuronal differentiation by measuring the thickness of NeuN^+^ layer. The results showed that BMP inhibitors ameliorated the SMG‐induced neuronal number shortage (Figure 3M,N). In conclusion, these data indicate that SMG‐induced upregulation of BMP signaling plays a crucial role in SMG‐mediated neuropathological defects.

### 2.4 Knockdown of ID1 Reverses SMG‐Mediated Defects

**Figure 4:**
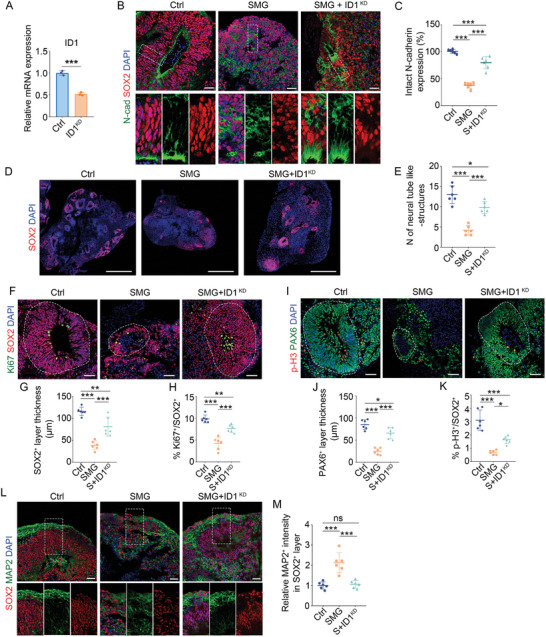
Knockdown of ID1 reverses SMG‐mediated defects. A) qRT‐PCR analysis of ID1 mRNA level in hESC H9 lines, demonstrating knockdown efficiency of shRNA‐ID1. *n* = 3 independent experiments. B,C) Immunostaining and quantification of the N‐cadherin expression in control (Ctrl), SMG‐treated (SMG), and knockdown of ID1 under SMG conditions (SMG+ID1^KD^) organoids at day 35. *n* = 6 individual organoids. D,E) Immunostaining and quantification of the number of neural tube‐like structures per 20 µm‐thick organoid section. F–K) Immunostaining and quantification of the thickness of SOX2^+^ and PAX6^+^ layer, and the cell proportion of Ki67^+^ and p‐H3^+^ proliferating cells. *n* = 6 individual organoids. L,M) Immunostaining and quantification of the proportion of MAP2^+^ neurons in SOX2^+^ layer. *n* = 6 individual organoids. Scale bar, 500 µm (D), 50 µm (B, F, I, L). Values were mean ± SD. Statistical significance was determined by unpaired, two‐tailed Student's *t*‐test (A). Statistical significance was determined by one‐way ANOVA with the Dunnet post hoc test (C, E, G, H, J, K, M). ns: *P* > 0.05, *p < 0.05, **p < 0.01, ***p < 0.001.

As ID1 expression was increased under SMG conditions, as assessed by bulk RNA‐seq and qRT‐PCR (Figure 2D,G), we hypothesized that the neuropathological defects in SMG‐treated brain organoids were attributable to increased expression of ID1. To test this hypothesis, we established ID1‐knockdown (ID1‐KD) organoids (**Figure** **4**A). Immunostaining results showed that the expression of N‐cadherin and the number of neural tube‐like structures were significantly rescued in SMG‐treated organoids after knockdown of ID1 (Figure 4B–E). Furthermore, the reduced proliferative capacity and thickness of NSPCs layer in SMG‐treated organoids were partially restored in ID1‐KD groups (Figure 4F–K). These results demonstrated that the SMG‐induced defects could be rescued by regulating the ID1 gene. Importantly, mis‐positioning of MAP2^+^ neurons was stronger in SOX2^+^ NSPCs in SMG‐treated organoids compared to control organoids, whereas knockdown of ID1 reduced the ectopic MAP2^+^ neuron expression (Figure 4L,M). These results demonstrate that ID1‐targeted intervention may be a potential strategy for treating SMG‐related neurodevelopmental defects.

### 2.5 YAP Activation Alleviates SMG‐Mediated Defects

**Figure 5:**
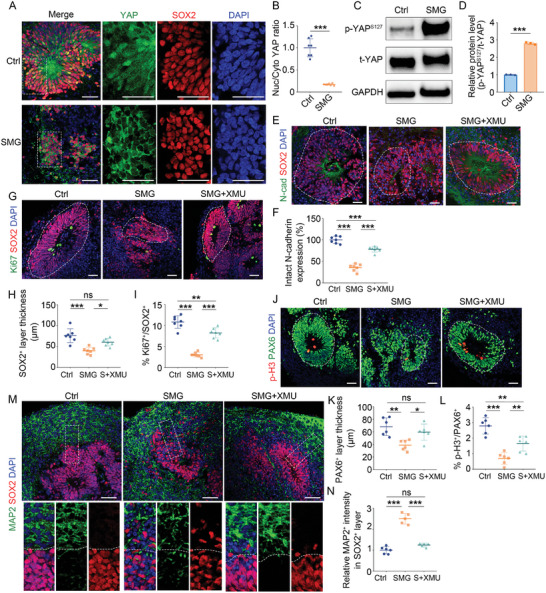
YAP activation alleviates SMG‐mediated defects. A,B) Fluorescence images and quantification of the ratio of YAP between nuclear and cytoplasmic intensity in NSPCs in control and SMG‐treated organoids on day 35. *n* = 7 individual organoids. C,D) Representative western blots of phosphorylated YAP protein levels in control and SMG‐treated groups on day 35. *n* = 3 independent experiments. E,F) Immunostaining and quantification of the N‐cadherin expression in control (Ctrl), SMG‐treated (SMG), and XMU‐treated under SMG conditions (SMG+XMU) organoids at day 35. *n* = 7 individual organoids. G–L) Immunostaining and quantification of the cell proportion of Ki67^+^ and p‐H3^+^ proliferating cells, and the thickness of SOX2^+^ or PAX6^+^ layer. M,N) Immunostaining and quantification of the ectopic neurogenesis in VZ. *n* = 6 individual organoids. Scale bar, 50 µm. Values were mean ± SD. Statistical significance was determined by Mann‐Whitney test (B). Statistical significance was determined by unpaired, two‐tailed Student's *t*‐test (D). Statistical significance was determined by one‐way ANOVA with the Dunnet post hoc test (F, H, I, K, L, N). ns: *P* > 0.05, *p < 0.05, **p < 0.01, ***p < 0.001.

Previous studies have revealed that YAP plays an important role in neural tube patterning by translating the physical cues in molecular terms.^[^ ^35^ ^]^ As our RNA‐Seq analyses showed the decrease of Hippo pathway signature genes in SMG‐treated organoids, we explored the effects of YAP on SMG‐treated organoid development. To determine the subcellular localization of YAP in organoids, we performed immunofluorescence. YAP was clearly cytoplasmic in NSPCs from SMG‐treated organoids but became predominately nuclear in control organoids (**Figure** **5**A,B). As YAP is regulated by the Hippo pathway through its phosphorylation,^[^ ^28^ ^]^ we performed western blot analysis. We observed higher expression of YAP phosphorylation on serine 127 under SMG conditions (Figure 5C,D). Collectively, these data indicate that YAP activity and nuclear localization are deceased by SMG treatment.

As nuclear YAP is required for AJs regulation,^[^ ^36^ ^]^ we determined whether increasing YAP activity could rescue the deficits in AJs formation. We utilized a selective MST1/2 inhibitor, XMU‐MP‐1 (XMU), which promotes nuclear localization and activation of YAP.^[^ ^43^ ^]^ Since YAP has been shown to promote tumor growth and metastasis,^[^ ^44^, ^45^ ^]^ we tested the appropriate dose of XMU in brain organoid development. Based on the light images and immunostaining results, no impairment of brain organoid growth was observed at the dose of 0.03 µM, which exerted an apparent nucleation pharmacological effect (Figure S3A–H, Supporting Information). This dose was used for subsequent intervention under SMG conditions. We found that the expression of N‐cadherin and the number of neural tube‐like structures in SMG‐treated organoids were increased after XMU treatment (Figure 5E,F; Figure S3I,J, Supporting Information). Furthermore, XMU improved the proliferative capacity of NSPCs and the thickness of NSPCs layer in SMG‐treated organoids (Figure 5G–L). The ectopic neurogenesis of SMG‐treated organoids was rescued by the use of XMU (Figure 5M,N). These results imply that YAP is responsive to SMG, leading to impaired AJs formation, which in turn determines NSPCs proliferation and differentiation as well as tissue morphogenesis.

### 2.6 Loss of YAP Results in an Increase in BMP Signaling

**Figure 6:**
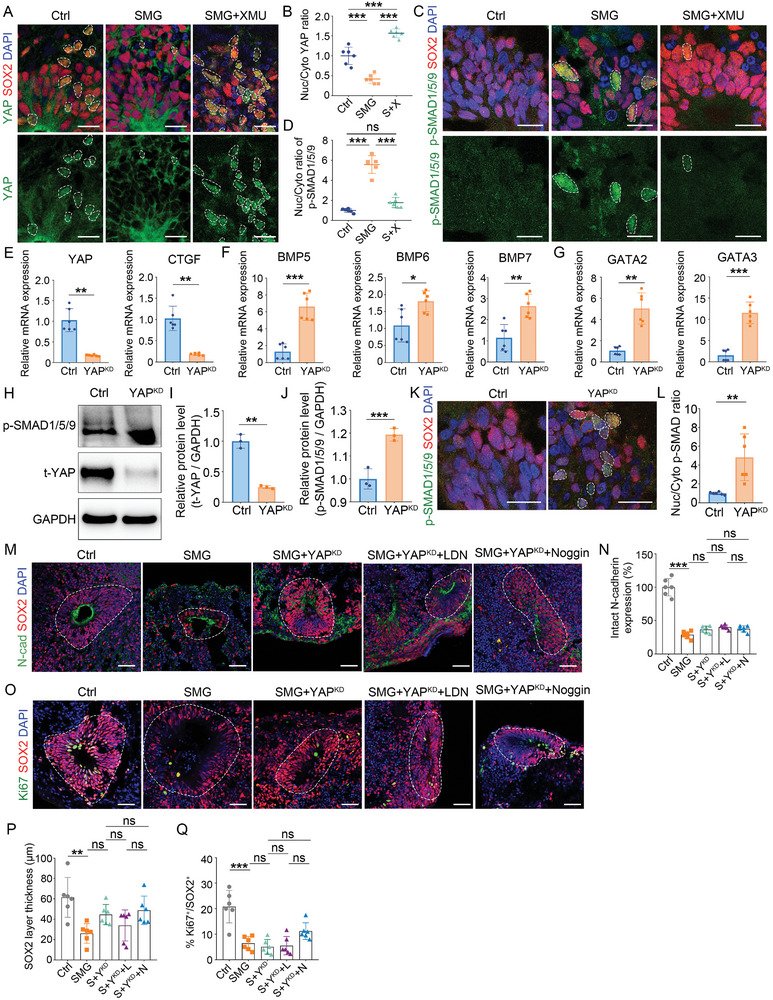
Loss of YAP results in an increase in BMP signaling. A,B) Fluorescence images and quantification of the ratio of YAP between nuclear and cytoplasmic intensity in NSPCs in control, SMG, and SMG+XMU groups on day 35. *n* = 6 individual organoids. C,D) Fluorescence images and quantification of the ratio of phosphorylated SMAD1/5/9 between nuclear and cytoplasmic intensity in NSPCs in control, SMG, and SMG+XMU groups on day 35. *n* = 6 individual organoids. E–G) The relative mRNA expression level of *YAP* gene, YAP target gene, BMPs, and BMP target genes in control and YAP‐knockdown (YAP^KD^) organoids at day 35*. n* = 6 independent experiments. H–J) Representative western blots and quantification of phosphorylated SMAD1/5/9 and total YAP protein levels. *n* = 3 independent experiments. K,L) Fluorescence images and quantification of the ratio of phosphorylated SMAD1/5/9 between nuclear and cytoplasmic intensity in NSPCs in control and YAP^KD^ organoids at day 35. *n* = 6 individual organoids. M,N) Immunostaining and quantification of the expression of N‐cadherin in control, SMG‐treated, YAP‐knockdown under SMG conditions (S+Y^KD^), YAP‐knockdown with LDN193189 under SMG conditions (S+Y^KD^+L), and YAP‐knockdown with Noggin under SMG conditions (S+Y^KD^+N) organoids at day 35. *n* = 6 individual organoids. O–Q) Immunostaining and quantification of the thickness of SOX2^+^ layers, and the cell proportion of Ki67^+^ proliferating cells in SOX2^+^ NSPCs. *n* = 6 individual organoids. Scale bar, 20 µm (A, C, K), 50 µm (M, O). Values were mean ± SD. Statistical significance was determined by unpaired, two‐tailed Student's *t*‐test (E, F, G, I, J, L). Statistical significance was determined by one‐way ANOVA with the Dunnet post hoc test (B, D, N, P, Q). ns: *P* > 0.05, *p < 0.05, **p < 0.01, ***p < 0.001.

YAP/TAZ can integrate mechanical signals with BMP signaling to maintain AJs compliance and integrity in angiogenic vessels.^[^ ^36^ ^]^ However, whether YAP integrates mechanical signals with BMP signals in the neural tube has not been fully demonstrated. To gain insight into the relationship between BMP signaling and nuclear YAP in neural tube, we immunostained XMU‐treated organoids for YAP and p‐SMAD1/5/9. Immunostaining of organoids treated with XMU revealed increased YAP nuclear localization and decreased p‐SMAD1/5/9 nuclear expression (**Figure** **6**A–D). To elucidate the transcriptional and translational targets of YAP, we generated YAP‐knockdown brain organoids, as previously reported.^[^ ^46^ ^]^ As expected, knockdown of YAP resulted in reduced gene expression of both *YAP* and the YAP target gene (*CTGF*) (Figure 6E). Interestingly, YAP knockdown promoted the expression of the *BMPs* and BMP target genes (Figure 6F,G). Furthermore, YAP knockdown significantly increased the abundance and nuclear localization of p‐SMAD1/5/9, as detected by western blot analysis and immunostaining (Figure 6H–L). These results indicate that YAP activation inhibits BMP signaling in brain organoids.

We then hypothesized that BMP activation is the downstream of YAP inhibition in brain organoids. To test this hypothesis, we used BMP inhibitors to rescue the effects of YAP knockdown. The results showed that BMP inhibitors were effective in ameliorating the defects of YAP‐knockdown brain organoids, specifically in the formation of AJs, proliferation and maintenance of NPSCs, and neuron differentiation (Figure S4A–G, Supporting Information).

To address whether YAP was responsible for increasing BMP signaling under SMG conditions, we used BMP inhibitors to manipulate BMP signaling in YAP knockdown organoids under SMG conditions. Strikingly, the AJs formation and the number of neural tube‐like structures were not affected by the BMP inhibitors (Figure 6M,N; Figure S4H,I, Supporting Information). There was also no difference in the proliferative capacity of NSPCs and the thickness of NSPCs layer after suppressing BMP signaling in YAP knockdown organoids under SMG conditions (Figure 6O–P). To help rule out off‐target effects, YAP was re‐expressed in YAP‐knockdown organoids followed by BMP inhibitors treatment. The results showed that the BMP inhibitors were effective in alleviating the SMG‐induced defects by re‐expressing YAP in YAP‐knockdown organoids (Figure S5, Supporting Information). As previous studies have reported that YAP supports Smad1‐dependent transcription and is required for BMP inhibition of neural differentiation^[^ ^47^ ^]^ and NSPCs proliferation,^[^ ^48^ ^]^ our study also found that a severe deficiency of YAP resulted in the inability of BMP inhibitors to rescue the growth of brain organoids under SMG conditions. These results indicate that YAP plays a crucial role not only in translating mechanical signals into BMP signaling during neural tube morphogenesis, but also supporting the activation of BMP signaling.

### 2.7 Single‐Cell RNA‐Seq Reveals Dysregulated NSPCs Clusters in SMG‐Treated Organoids

**Figure 7:**
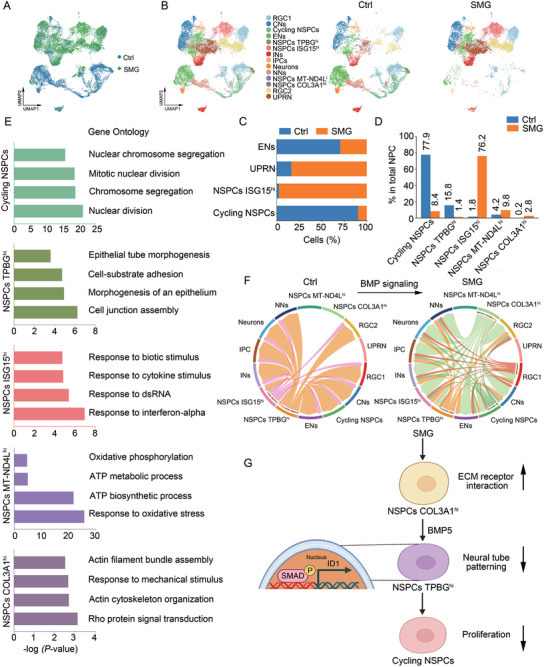
Single‐cell RNA‐Seq reveals dysregulated NSPCs clusters in SMG‐treated organoids. A,B) Uniform manifold approximation and projection (UMAP) plots of brain organoids‐derived single cells distinguished by treatment methods (A) or annotations (B). C) Relative proportions of cells in the major clusters between the control and SMG‐treated organoids. D) Proportions of different types of NSPCs among total NSPCs. E) Histograms of the four top GO terms of NSPCs. Color coding of bars represent cluster identities. F) Chord plot of the BMP signaling interaction network among all clusters in control and SMG‐treated groups. G) Diagram of the mechanisms of SMG. RGCs, radial glia cells; CNs, cortical neurons; ENs, excitatory neuron; NSPCs, neural stem and progenitor cells; INs, interneurons; IPC, intermediate progenitor cells; NNs, new‐born neurons; UPRN, unfolded protein response‐related neurons.

To gain insight into the cell‐type‐specific changes in SMG‐treated organiods, single‐cell RNA sequencing (scRNA‐seq) was performed in control and SMG‐treated organoids on day 35. After quality control and filtering, unbiased clustering identified 23 clusters based on transcript signatures. These clusters were further categorized into 14 cell types according to the expression of known cell‐type markers in combination with GO analysis (**Figure** **7**A,B; Figure S6A–C, Supporting Information).

To better understand the changes in NSPCs subtypes, NSPCs were further defined into 5 subtypes by distinct upregulated gene signatures in each cluster (Figure S6D, Supporting Information). The subpopulation of NSPCs were referred as “TPBG^hi^”, “ISG15^hi^”, “MT‐ND4L^hi^”, “COL3A1^hi^”, and cycling NSPCs. Compared to control samples, an apprarent increased proportion of unfolded protein response‐related neurons (UPRN) and ISG15^hi^ NSPCs in SMG‐treated organoids was observed, alongside a decreased proportion of excitatory neurons (ENs) and cycling NSPCs (Figure 7C). In NSPCs, the cycling NSPCs and TPBG^hi^ NSPCs were enriched in control samples, while the abundance of ISG15^hi^ NSPCs, MT‐ND4L^hi^ NSPCs, and COL3A1^hi^ NSPCs were increased in SMG‐treated samples (Figure 7D). Subsequently, we investigated the characteristics of each NSPCs cluster. The results showed that cycling NSPCs cluster was enriched for genes involved in nuclear division and chromosome segregation. The upregulated genes in TPBG^hi^ NSPCs cluster were involved in cell junction assembly, morphogenesis of an epithelium, and epithelial tube morphogenesis. Intriguingly, the COL3A1^hi^ NSPCs cluster was enriched for genes in the signatures of actin cytoskeleton organization and response to mechanical stimulus (Figure 7E). Collectively, SMG increases the number of COL3A1^hi^ NSPCs, which are responsible for translating mechanical signals. Furthermore, SMG decreases the number of cycling NSPCs and TPBG^hi^ NSPCs, which are predominantly enriched for cell junction assembly and tube morphogenesis.

To further elucidate the biological functions of COL3A1^hi^ NSPCs and TPBG^hi^ NSPCs, Gene Set Enrichment Analysis (GSEA) was performed in control and SMG‐treated organoids. GSEA revealed that the enrichment of ECM receptor interaction and integrin mediated signaling pathway were upregulated in COL3A1^hi^ NSPCs after SMG treatment (Figure S6E, Supporting Information). As ECM provides cells with mechanical cues,^[^ ^49^ ^]^ these results indicate that enhanced function and increased number of COL3A1^hi^ NSPCs may help translate microgravity cues into molecular terms. Moreover, the rostrocaudal neural tube patterning genes were downregulated by SMG treatment in TPBG^hi^ NSPCs (Figure S6F, Supporting Information). These findings suggest that impaired function and reduced number of TPBG^hi^ NSPCs under SMG conditions may lead to AJs formation deficits. These data elucidate the SMG‐induced phenotypes in brain organoids at the single‐cell level and emphasize the crucial role of COL3A1^hi^ NSPCs, TPBG^hi^ NSPCs, and cycling NSPCs in SMG‐induced defects.

To expolore the differences of cell cluster interactions in control and SMG‐treated organoids, we performed cell–cell communication analysis (Figure S7A–C, Supporting Information). We observed a strong increase in the interactions between COL3A1^hi^ NSPCs and other cell clusters in BMP signaling (Figure 7F). The expression of BMP ligand and receptor genes in each cluster was further analyzed. BMP5 was specifically enriched in COL3A1^hi^ NSPCs, while BMP receptor genes were high expressed in all NSPCs clusters, except for MT‐ND4L^hi^ NSPCs (Figure S7D, Supporting Information). Together, these results suggest that COL3A1^hi^ NSPCs may act as a mechanosensitive cell clutser that translating microgravity cues into BMP5, which then activates BMP signaling in TPBG^hi^ NSPCs. BMP activation impedes the AJs formation in TPBG^hi^ NSPCs, leading to a reduction in cycling NSPCs (Figure 7G).

Space microgravity has been reported to enhance stress pathways and energy production of NSPCs.^[^ ^50^, ^51^, ^52^ ^]^ Oxidative stress represents a key driver of numerous physio‐pathological events, including inflammatory responses^[^ ^53^ ^]^ and cellular apoptosis.^[^ ^54^ ^]^ Our scRNA‐seq data showed an increase in the number of MT‐ND4L^hi^ NSPCs associated with oxidative stress and adenosine triphosphate (ATP) metabolic process under SMG conditions. High metabolic demands and oxidative stress always induce endoplasmic reticulum stress and activation of the unfolded protein response (UPR).^[^ ^55^, ^56^ ^]^ In our study, the proportion of UPRN was increased after SMG treatment. To investigate whether the inflammatory response and apoptosis were elevated in SMG‐treated organoids, we performed qRT‐PCR analysis of pro‐inflammatory cytokine expression and TUNEL staining for apoptosis. The results showed that the expression of pro‐inflammatory cytokine was significantly increased in SMG‐treated organoids (Figure S8A, Supporting Information). Furthermore, SMG induced an increase in the proportion of TUNEL^+^ cells to total cells (Figure S8B, Supporting Information). Taken together, our study provides evidence that stress pathways are increased in both neurons and NSPCs after SMG treatment, further highlighting the influence of SMG in stress pathways.

### 2.8 SMG Intervention Leads to Persistent Neurologic Damage

Previous studies have reported that the changes in NSPCs exposed to microgravity are not permanent, since the return to normal gravity restores the original characteristics of NSPCs.^[^ ^57^ ^]^ Nevertheless, some other studies support that the microgravity effects remain even after returning to normal gravity conditions.^[^ ^58^ ^]^ However, it is unclear whether SMG‐treated neuropathological defects persist after a return to normal gravity.

To gain insight into the effects of SMG intervention at different time points on neurodevelopment, we exposed brain organoids at different culture time periods, including initiation at the neural induction (NI; D0), neural differentiation (ND; D10), and neural maturation (NM; D18) stage (Figure S9A, Supporting Information). SMG treatment initiated at different culture phases resulted in impaired AJs formation (Figure S9B, Supporting Information). Moreover, the proliferative capacity and maintenance of NSPCs were reduced to similar levels after different time periods of SMG treatment (Figure S9C–E, Supporting Information). A similar phenotype was even observed in the 4‐day SMG treatment (Figure S10A–C, Supporting Information). Intriguingly, the mis‐positioning of neurons in SOX2^+^ NSPCs was not affected by SMG treatment initiated at the NM stage, whereas it was abnormally enhanced by SMG treatment initiated at the ND stage and NI stage (Figure S9F,G, Supporting Information). This may be a result of the shorter duration of microgravity treatment. Furthermore, these results suggest a temporal sequence of SMG‐induced proliferative responses and ectopic neurogenesis.

**Figure 8:**
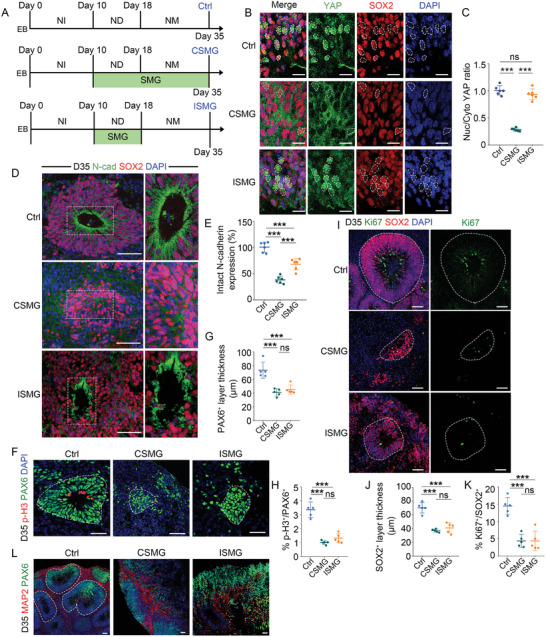
SMG intervention leads to persistent neurologic damage. A) Schematic of experimental design. NI, neural induction; ND, neural differentiation; NM, neural maturation; CSMG, continuous stimulated microgravity; ISMG, intermittent stimulated microgravity. B,C) Fluorescence images and quantification of the ratio of YAP between nuclear and cytoplasmic intensity in NSPCs in control, CSMG‐treated, and ISMG‐treated organoids. *n* = 6 individual organoids. D,E) Immunostaining and quantification of the expression of N‐cadherin. *n* = 6 individual organoids. F–K) Immunostaining and quantification of the thickness of SOX2^+^ or PAX6^+^ layers, and the cell proportion of Ki67^+^ or p‐H3^+^ proliferating cells in NSPCs. *n* = 5 individual organoids. L) Immunostaining of MAP2^+^ neurons and NSPCs. Scale bar, 20 µm (B), 50 µm (D, I, F, L). Values were mean ± SD. Statistical significance was determined by one‐way ANOVA with the Dunnet post hoc test. ns: *P* > 0.05, ***p < 0.001.

To explore whether microgravity interventions have sustained effects on neurodevelopment, we exposed brain organoids under SMG conditions initiated at ND stage, and returned them to normal gravity in NM stage, which was defined as intermittent SMG (ISMG). Exposing brain organoids under SMG conditions during the ND stage and NM stage was defined as continuous SMG (CSMG). Brain organoids cultured under normal gravity conditions were regarded as controls. We compared the growth of brain organoids in the control, CSMG, and ISMG group (**Figure** **8**A). Upon restoration of normal gravity, YAP subcellular localization in ISMG group returned to a level similar to those in the control group (Figure 8B,C), indicating that mechano‐sensitivity in SMG‐treated brain organoids was not impaired. The intact N‐cadherin expression and number of neural tube‐like structures in ISMG group were higher than that in the CSMG group, indicating that the restoration of normal gravity partially rescued the impaired AJs formation (Figure 8D,E; Figure S10D,E, Supporting Information). However, the impaired proliferative capacity, reduced number of NSPCs, and structural disorganization persisted after a return to normal gravity conditions on day 35 (Figure 8F–L). Moreover, we extended the recovery period post‐withdrawal to day 55. The thickness of MAP2^+^ layer in the ISMG group was significantly lower than that in the control group, suggesting the impairment of neuron differentiation (Figure S10F,G, Supporting Information). These results indicate that SMG intervention leads to persistent neurologic damages in brain organoids.

## 3 Discussion

In this study, we evaluated neurodevelopmental defects under SMG conditions using brain organoid models. SMG impaired AJs formation, leading to reduced proliferative capacity and maintenance of NSPCs, as well as ectopic neurogenesis. Transcriptomic results revealed that the BMP and YAP pathways may be responsible for these defects. Moreover, gene modulation and treatment with small‐molecule inhibitors targeting the YAP/BMP/ID1 axis rescued the SMG‐treated defects. scRNA‐seq results further identified dysregulated subpopulations of NSPCs, one of which translated SMG cues into BMP signals to the subpopulation responsible for the tube morphogenesis and ultimately regulated the proliferating cell population. Taken together, our findings precisely elucidate the critical role of YAP/BMP/ID1 axis in regulating NTDs in SMG‐treated organoids, and thus provide a new therapeutic strategy for weightlessness‐related neurodevelopmental defects.

In the embryonic nervous system, NSPCs generate their own self‐supportive niche through N‐cadherin‐based AJs contacts within the neuroepithelium.^[^ ^59^ ^]^ The AJs are a cell‐cell adhesion structure at the apical tip of NSPCs in the neural tube. A major function of AJs is to maintain the physical connections between NSPCs, as disruption of them contributes to loose cell‐cell contacts, resulting in neuroepithelial disorganization.^[^ ^40^ ^]^ Deficits in AJs formation lead to aberrant niche signaling that regulates NSPCs self‐renewal and cortical neurogenesis.^[^ ^39^, ^41^ ^]^ Recent findings highlight that mechanical forces drive AJs remodeling and polarization, thereby regulating the rate, pattern, and orientation of various cellular processes responsible for tissue size, shape, and organization.^[^ ^24^ ^]^ In our study, SMG impaired the N‐cadherin‐based AJs formation, resulting in impaired proliferation and disorganization in human brain organoids.

Neural tube patterning is mediated by morphogen signals.^[^ ^60^, ^61^ ^]^ The key process involves the formation of the median/ dorsolateral hinge points, around which the neural plates roll up and close to form the neural tube.^[^ ^62^ ^]^ BMP signaling has been reported to abolish hinge point formation and induces NTDs.^[^ ^18^, ^20^ ^]^ Inhibition of BMP signaling is required to maintain neural tube growth and morphology.^[^ ^18^, ^19^ ^]^ In our research, SMG induced the activation of BMP signaling. Inhibition of BMP signaling by suppressing the expression of the target gene *ID1* or using the BMP receptor inhibitors under SMG conditions restored neural tube formation.

Mechanical forces have been shown to feed into YAP/TAZ activity to mediate stem cell functions.^[^ ^27^ ^]^ YAP can translate mechanical stimuli into biochemical signals controlling organ size and tissue homeostasis.^[^ ^27^, ^28^ ^]^ Numerous studies have supported that YAP plays an important role in regulating the proliferation rate of NSPCs and neural tube patterning.^[^ ^29^, ^34^ ^]^ In our study, SMG treatment significantly decreased YAP activity. YAP activation could restore AJs formation and enhance NSPCs proliferative capacity under SMG conditions. These findings support that SMG impairs the growth and patterning of neural tube development through modulating YAP activity. YAP targeted therapies may be a potential strategy for treating SMG‐related neurodevelopmental defects. As our previous study reported a significant decrease in total YAP protein expression in brain organoids at day 55 compared to day 35 during normal development,^[^ ^46^ ^]^ the therapeutic effect of regulation of YAP activity may be closely related to the treatment periods. More evidence of efficacy can be available if the treatment duration is extended to day 55.

Previous studies have reported crosstalk between BMP signaling and YAP/TAZ, and the function of YAP on BMP signaling is currently controversial.^[^ ^36^, ^37^, ^38^ ^]^ Our results found that YAP activation inhibited BMP signaling in brain organoids, whereas knockdown of YAP significantly increased BMP signaling. Inhibition of BMP signaling was effectively alleviate NTDs after YAP knockdown under normal gravity, indicating that BMP activation is the downstream of YAP inhibition in brain organoids.

Previous studies have revealed that BMP‐induced phosphorylation of Smad 1 recruit nuclear YAP to *Id* genes for enhanced transcription.^[^ ^47^, ^63^, ^64^ ^]^ Moreover, the effect of BMP on the *Id* gene expression is sensitive to YAP depletion.^[^ ^47^ ^]^ In our study, inhibition of BMP signaling did not effectively alleviate NTDs after YAP knockdown under SMG conditions. We think that whether BMP inhibitors work under normal gravity or SMG condition is dependent on the level of nuclear YAP. Typically, YAP knockdown reduces the total YAP level, but has no significant effect on the p‐YAP/YAP ratio.^[^ ^65^ ^]^ Thus, a certain level of nuclear YAP is still present in the YAP knockdown brain organoids under normal gravity. However, SMG induced more YAP accumulation in the cytoplasm and reduced nuclear YAP levels compared to normal gravity. Further reduction of nuclear YAP in YAP knockdown brain organoids under SMG conditions may interfere with the effect of BMP inhibitors. The YAP knockout by CRISPR/Cas9‐mediated gene editing may provide more details of the mechanisms.

Brain organoids offer an unprecedented platform to study brain development under SMG conditions, which affect various cell types and complex cellular interactions. Our scRNA‐seq data identified a reduction in cycling NSPCs and TPBG^hi^ NSPCs, which are responsible for NSPCs proliferative capacity and neural tube morphogenesis, respectively. In addition to the decreased number of TPBG^hi^ NSPCs, the rostrocaudal neural tube patterning function of TPBG^hi^ NSPCs was impaired under SMG conditions. Intriguingly, the number of COL3A1^hi^ NSPCs exhibiting a mechanical stimulus‐sensitive phenotype increased after SMG treatment. Moreover, the ECM receptor interaction and integrin mediated signaling pathway were elevated in COL3A1^hi^ NSPCs after SMG treatment. This NSPCs cluster may be enriched with mechanosensors or gravireceptors that respond rapidly to changes in gravitational forces and connect to the cytoskeleton or ECM. These findings suggest that SMG induces neuropathological defects possbily by regulating the number and function of different NSPCs subpopulations.

Deciphering cell‐to‐cell cross‐talk in the brain will deepen our understanding of molecular disease pathophysiology.^[^ ^66^ ^]^ In addition to exploring cell‐type‐specific perturbations, our study elucidates cell‐cell interactions involved in BMP signaling under SMG treatment. COL3A1^hi^ NSPCs that specifically express BMP5 have increased interaction with other cell clusters in BMP signaling under SMG conditions. Previous reports have revealed that overexpression of integrins in the early neural tube selectively induces BMP5.^[^ ^67^ ^]^ Moreover, the ECM protein anosmin regulates BMP5 signaling during neural crest formation.^[^ ^68^ ^]^ Our results suggest that COL3A1^hi^ NSPCs may convert microgravity cues into BMP5. Deciphering the mechano‐transduction at the single‐cell level under SMG conditions can help target specific molecules. More work is needed to understand how BMP5 regulates neural tube formation under microgravity conditions.

Despite we observed detailed neurodevelopmental phenotypic changes in this study, these phenotypes are produced using SMG as a model system, which is unable to recapitulate the complex spatial environment.^[^ ^69^ ^]^ Further studies of brain organoids during in‐flight missions will refine the neurodevelopmental phenotypes and associated mechanisms in space. Moreover, integrating spatial transcriptomics, photoactivatable fluorescent reporters, and scRNA‐seq can provide additional information to elucidate cellular and molecular changes in brain organoids.^[^ ^66^ ^]^ Overall, our current work provides a resource to better understand the relationship between mechano‐transduction and neural tube development in microgravity. These results help develop effective countermeasures to normalize brain development in microgravity and spaceflight.

## 4 Experimental Section

### hPSCs Culture

Human embryonic stem cells (hESCs) (H9, WiCell Agreement No. 24‐W0484) were seeded onto 6‐well tissue culture plates precoated with Matrigel and maintained in mTeSR plus medium. Cells were fed daily and passaged every 4–5 days following treatment with 0.5 mM ethylene diamine tetraacetic acid (EDTA) solution for 8 min. Cells were routinely confirmed to be negative for mycoplasma with the MycoAlert Kit (Lonza).

### Generation of ID1 Knockdown hESC Lines

At ≈40% confluence, the hESCs were incubated with lentiviruses for 12 h. ID1 shRNA targeting sequences were used as followed: CTACGACATGAACGGCTGTTA. Next, puromycin was added to the cell cultures for 2 weeks at increasing concentrations from 0.05 µg mL^−1^ to 0.5 µg mL^−1^. Cells were then dissociated into single cells by Accutase treatment for 10 min at 37 °C, and transferred to a flat bottom 96‐well plate (1 cell/well) containing medium supplemented with 5 µM Y27632. After a week, single clones were picked and expanded for 2 weeks.

### YAP Knockdown and Re‐Expression

For generation of YAP knockdown hESC lines, short hairpin RNA (shRNA) YAP‐expressing lentiviruses were purchased from Obio Technology (Shanghai, China). The targeting sequences were as follows: ShRNA 1, 5′‐CAGGTGATACTATCAACCAAA‐3′; ShRNA 2, 5′‐GACCAATAGCTCAGATCCTTT‐3′; ShRNA 3, 5′‐CCACCAAGCTAGATAAAGAAA‐3′. The detailed methods for the transfection and extraction of individual clones from hESCs were similar to those used to generate the ID1 knockdown cell lines. For YAP re‐expression studies, individual YAP knockdown organoid was incubated with 1 µL 10^8^ TU mL^−1^ lentiviruses to overexpress YAP in 200 µL neural mature medium for 12 h on day 18. After co‐incubation, the organoids were treated with BMP inhibitors under SMG conditions.

### Generation of Brain Organoids

Human brain organoids were generated as described previously.^[^ ^70^, ^72^ ^]^ In brief, ≈9000 single cells in neural induction medium were plated in ultra‐low‐attachment 96‐well plates to form embryoid bodies (EBs). The neural induction medium was prepared as follows: DMEM/F12 media, 15% [v/v] KSR, 1% [v/v] GlutaMAX, 1% [v/v] MEM‐NEAA, 100 µM β‐mercaptoethanol, 100 nM LDN‐193189, 10 µM SB‐431542, 2 µM XAV‐939, and 50 µM Y27632. On day 10, organoids were transferred into ultra‐low‐attachment 6‐well plates in neural differentiation medium containing 50% DMEM‐F12, 50% Neurobasal medium, 0.5% [v/v] N2 supplement, 1% [v/v] B27 supplement minus vitamin A, 0.025% [v/v] insulin, 0.5% [v/v] MEM‐NEAA, 1% [v/v] Glutamax, 1% [v/v] Penicillin/Streptomycin, 50 µM β‐Mercaptoethanol. Organoids were cultured on an orbital shaker at 80 rpm min^−1^. On day 18, organoids were maintained in neural mature medium comprising 50% DMEM‐F12, 50% Neurobasal medium, 0.5% (v/v) N2 supplement, 1% (v/v) B27 supplement, 0.5% (v/v) MEM‐NEAA, 1% (v/v) Glutamax, 0.025% (v/v) Insulin, 50 µM β‐Mercaptoethanol, 1% (v/v) Penicillin/Streptomycin, 20 ng mL^−1^ BDNF, and 200 mM ascorbic acid. The medium was refreshed every 4 days.

### Simulated Microgravity

A 3D clinostat (SM‐31, Center for Space Science and Applied Research, Chinese Academy of Sciences), which was also called a random positioning machine, was used to simulate microgravity (SMG). The clinostat contains two axes and the rotations of the two frames were driven by separate motors. The rotation of each frame was random and autonomous by a controller. The SMG was generated by averaging the gravity vector over the independent rotation of the two frames. According to previous publications^[^ ^73^, ^74^, ^75^ ^]^ and patent (A method for evaluating microgravity biaxial rotators, Patent No. CN101726426B), setting a random rotating speed of 0 to 10 rpm could establish 10^−3^ G conditions in 5 min, and it was defined as simulated microgravity (10^−3^ G).

In this study, EB at day 0 formed in ultra‐low‐attachment 96‐well plates was transferred to T25 cell culture flasks. The flasks were filled with neural induction medium to reduce the interference of liquid shear forces, and sealed with parafilm to prevent liquid leakage contamination. On day 10, the organoids were transferred onto 35 mm cell culture dishes filled with neural differentiation medium to reduce media usage. On day 18, the organoids were cultured in 35 mm cell culture dishes filled with neural mature medium. The medium was refreshed every 4 days. The cell culture flasks or dishes were fixed to the clinostat, which was set in the random speed of 0 to 10 rpm. The clinostat was placed in a humidified 37 °C incubator with 5% CO_2_ for the duration of the experiments.

### Drug treatment

The lack of apparent ventricles in SMG‐induced organoids at day 55 makes it difficult to recognize the formation of AJs attached to the surface of the ventricular lumen. Day 35 was therefore chose as the treatment endpoint in the majority of our experiments to directly evaluate the AJs formation. Organoids were treated with 1 µM LDN‐193189, or 100 ng mL^−1^ Noggin, or XMU. The BMP inhibitor treatments (LDN‐193189 or Noggin) started on day 18 of the protocol and continued through until day 35 or day 55 with fresh drug supplementation every 4 days during medium change. The XMU treatments started on day 18 of the protocol and continued through until day 35 with fresh drug supplementation every 4 days during medium change.

### Cryo‐Section and Immunostaining of Organoids

Brain organoids were collected on days 35 (period of extensive proliferative NSPCs production) and days 55 (period of extensive neurons production), as previously described.^[^ ^70^, ^71^ ^]^ In brief, organoids were fixed in 4% paraformaldehyde (PFA) in phosphate‐buffered saline (PBS) overnight at 4 °C. After washing in PBS, organoids were transferred to a 30% sucrose solution for 1 day at 4 °C. After embedding in O.C.T compound on dry ice, organoids were sectioned with a cryostat (CM3050 S, Leica) at 20 µm. The slices were incubated with 0.1% Triton X‐100 in PBS for 15 min. After washing with PBS, sections were blocked with 10% goat serum in PBS for 1 h at room temperature. Slices were incubated at 4 °C overnight in primary antibodies diluted in 10% goat serum. The next day, sections were washed with PBS, and then incubated for 1 h at room temperature with secondary antibodies diluted in PBS. Finally, slices were stained by 4,6‐diamino‐2‐phenyl indole (DAPI) and mounted in ProLong Gold Antifade Reagent. Cell death was detected using the TUNEL assay according to the manufacturer's instructions (Thermo Fisher Scientific). Images were captured using a microscope (A1HD25, Nikon). To quantify the number of positive cells for each marker and the length of the VZ and CP layers, at least five randomized images were measured by a blinded counter using Fiji software. The primary antibodies were listed in Table S1 (Supporting Information).

### Western Blot Analysis

Organoids were lysed with radio immunoprecipitation assay lysis buffer (RIPA) containing a protease/phosphatase inhibitor cocktail. Twenty micrograms of total proteins were loaded for every sample. Samples were run on sodium dodecyl sulfate‐polyacrylamide gel electrophoresis and transferred to polyvinylidene fluoride membranes. Membranes were blocked in 5% BSA for 2 h at room temperature and incubated with primary antibodies diluted in 5% BSA overnight at 4 °C. Membranes were washed in TBST (Tris‐buffered saline, 0.1% Tween‐20 detergent) buffer before incubation with horseradish peroxidase‐labeled secondary antibodies diluted in 5% skim milk and visualized using an ECL system. Fiji software was used for western blot quantification. The primary antibodies were listed in Table S2 (Supporting Information).

### qRT‐PCR Analysis

Total RNA was extracted using the EZ‐press RNA purification kit. cDNA was produced using the RevertAid First Strand cDNA Synthesis Kit. qRT‐PCR was performed using the SYBR Green Master Mix. The relative expression in each sample was quantified using the 2^−ΔΔCt^ method and normalized using an endogenous reference glyceraldehyde‐3‐phosphate dehydrogenase (*GAPDH*) expression. The primers were listed in Table S3 (Supporting Information).

### scRNA‐Sequencing Library Preparation

The control and SMG‐treated organoids were prepared for scRNA sequencing (scRNA‐seq) on day 35. Briefly, three cortical organoids were dissociated into a single‐cell suspension via incubation with 1 mL of tryp‐LE in a 37 °C water bath for 30 min with gentle agitation every 5 min, followed by washed with 2% fetal bovine serum in DPBS. Approximately 10 000 cells in each group were loaded to chromium single‐cell 3 Chip and Illumina NovaSeq. Briefly, the captured cells were barcoded in individual gel beads in emulsions (GEMs). scRNA‐Seq libraries were generated using the Chromium Single Cell 3′ Library & Gel Bead Kit V3, and the resulting single‐cell libraries were sequenced using the Illumina NovaSeq 6000. The single cell sequence reads were mapped to the GRCh38 human genome. The number of median genes detected per cell was >3500, and the count of the median unique molecular identifier (UMI) per cell was >8000.

### scRNA‐Sequencing Data Analysis

The expression matrices processed with Seurat were summarized by the top 23 principal components. The transcriptomic profiles were visualized using UMAP. Cell clusters were annotated using unique cell type‐specific marker expression and GO enrichment.^[^ ^70^ ^]^ First, the 23 clusters were separated into eleven non‐neuronal cell, ten neuronal, and two intermediate cell clusters based on the expression patterns of early neurogenesis genes (*NES, NOTCH1*, *SOX2, HES1, ID1, ID3*) and neuronal growth cone genes (*GAP43, STMN2*, and *DCX*). Non‐neuronal clusters with enrichment of “cell cycle” and specific expression of *ASPM*, *MKI67*, and *TOP2A* were annotated as cycling NSPCs. RGCs was extracted by the enrichment of “‘glial cell differentiation”’. Among the cells with neuronal identity, it identified interneurons (*GAD1*, *GAD2*), excitatory neurons (*SLC17A6*), cortical neurons (*TBR1*), unfolded protein response‐related neurons (the enrichment of “endoplasmic reticulum unfolded protein response”). The cluster of intermediate progenitor cells (IPC) was extracted by the expression of *EOMES*, and the other intermediate cell cluster was defined as new‐born neurons. Cell‐to‐cell interactions based on the expression of known ligand–receptor pairs were predicted using CellChat. RGCs, radial glia cells; CNs, cortical neurons; ENs, excitatory neuron; NSPCs, neural progenitor cells; INs, interneurons; IPC, intermediate progenitor cells; NNs, new‐born neurons; UPRN, unfolded protein response‐related neurons.

### Bulk RNA‐Sequencing and Analysis

Total RNA from control and SMG‐treated organoids on days 35 was extracted using an EZ‐press RNA purification kit. RNA integrity was assessed using the RNA Nano 6000 Assay Kit on a Bioanalyzer 2100 system (Agilent Technologies). Sequencing libraries were generated on the Illumina NovaSeq platform. The human reference genome assembly (GRCh38) was used to map RNA‐Seq reads using HISAT2 software. Differential expression analysis of control and SMG‐treated organoids was performed using the DESeq2 R package. An absolute fold change of 2 and adjusted *P* value of 0.05 were used as thresholds.

### Quantification and Statistical Analyses

All data represent mean ± standard deviation (SD) unless otherwise stated. Statistical analyses were performed using the GraphPad Prism 8 software. Comparisons between two groups were performed using an unpaired two‐tailed Student's *t*‐test. Comparisons among multiple groups were performed using one‐way analysis of variance (ANOVA) with Dunnett post hoc test. Statistical significance was set at *P* < 0.05.

## Conflict of Interest

The authors declare no conflict of interest.

## Author Contributions

D.G., B.Y., W.‐W.S. contributed equally to this work. G.D., Y.B., and S.W.W. performed all experiments and wrote the manuscript. G.D., Y.B., and S.W.W. generated hESC lines, performed cellular, molecular and histological assays, and analyzed the data. Z.J.C., C.Z.H., and S.J.X. analyzed the scRNA‐seq data. H.N., B.S.Q., C.M.M. and F.X. assisted with the pharmacological treatments. L.X.H. supervised the project.

## Supporting information



Supporting Information

## Data Availability

The data that support the findings of this study are available in the supplementary material of this article.
