# Supplementary material for: The Critical Role of YAP/BMP/ID1 Axis on Simulated Microgravity‐Induced Neural Tube Defects in Human Brain Organoids
Source: Adv Sci (Weinh). 2024 Dec 10;12(5):2410188. doi: 10.1002/advs.202410188 (PMC11792043; doi:10.1002/advs.202410188)
Supplement: Supplementary file 1 — Supporting Information [file ADVS-12-2410188-s001.pdf]

ADVANCED SCIENCE
Open Access

# Supporting Information

for *Adv. Sci.*, DOI 10.1002/advs.202410188

The Critical Role of YAP/BMP/ID1 Axis on Simulated Microgravity-Induced Neural Tube Defects in Human Brain Organoids

*Di Guo, Bin Yao, Wen-Wei Shao, Jia-Chen Zuo, Zhe-Han Chang, Jian-Xin Shi, Nan Hu, Shuang-Qing Bao, Meng-Meng Chen, Xiu Fan and Xiao-Hong Li**

# The Critical Role of YAP/BMP/ID1 Axis on Simulated Microgravity-Induced Neural Tube Defects in Human Brain Organoids

Di Guo[1,2,3#], Bin Yao[1,2,3#], Wen-Wei Shao[1,2,3#], Jia-Chen Zuo[1,2,3], Zhe-Han Chang[1,2,3], Jian-Xin Shi[1,2,3], Nan Hu[1,2,3], Shuang-Qing Bao[1,2,3], Meng-Meng Chen[1,2,3], Xiu Fan[1,2,3], Xiao-Hong Li[1,2,3*]

[1]Academy of Medical Engineering and Translational Medicine, Tianjin University, Tianjin 300072, China

[2]State Key Laboratory of Advanced Medical Materials and Devices, Tianjin 300072, China

[3]Haihe Laboratory of Brain-Computer Interaction and Human-Machine Integration, Tianjin 300072, China

[#]Co-first author

*Correspondence: xhli18@tju.edu.cn

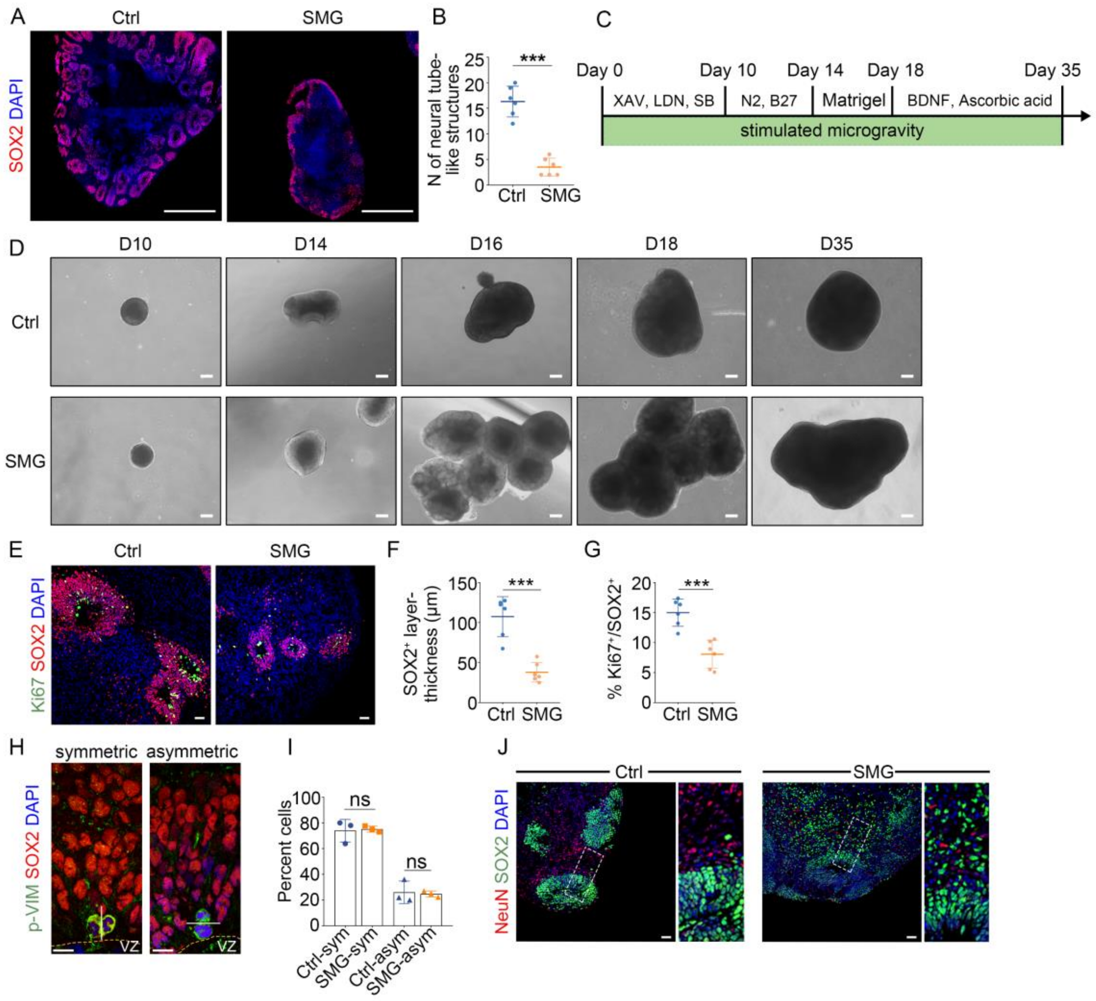


**Figure S1. Stimulated microgravity causes NTDs associated with impaired proliferative capacity and structural disorganization in brain organoids.** (A, B) Immunostaining and quantification of the number of neural tube-like structures per 20 μm-thick organoid section. *n* = 6 individual organoids. (C) Schematic of experimental design. The Matrigel was added to the medium from day 14 to day 18. (D) Light images of control or SMG-treated organoids from day 10 to day 35. The SMG-treated organoids were spontaneous fused once Matrigel was added. (E–G) Immunostaining and quantification of the thickness of $SOX2^+$ layer, and the cell proportion of $Ki67^+$ proliferating cells in control and SMG-treated organoids cultured in Matrigel-containing medium at day 35. *n* = 6 organoids. (H, I) Immunostaining and quantification of the percentage of asymmetric and symmetric division of NSPCs in the VZ-like regions. Yellow dotted lines indicate apical surface. White lines indicate the division plate. *n* = 3 individual organoids. (J) Immunostaining of the relative $NeuN^+$ intensity in $SOX2^+$ layer at day 35. Scale bar, 500 μm (A), 200 μm (D), 50 μm (E, J), 10 μm (H). Values were mean ± SD. Statistical significance was determined by unpaired, two-tailed Student's t-test. ns: $P > 0.05$, ***$P < 0.001$.

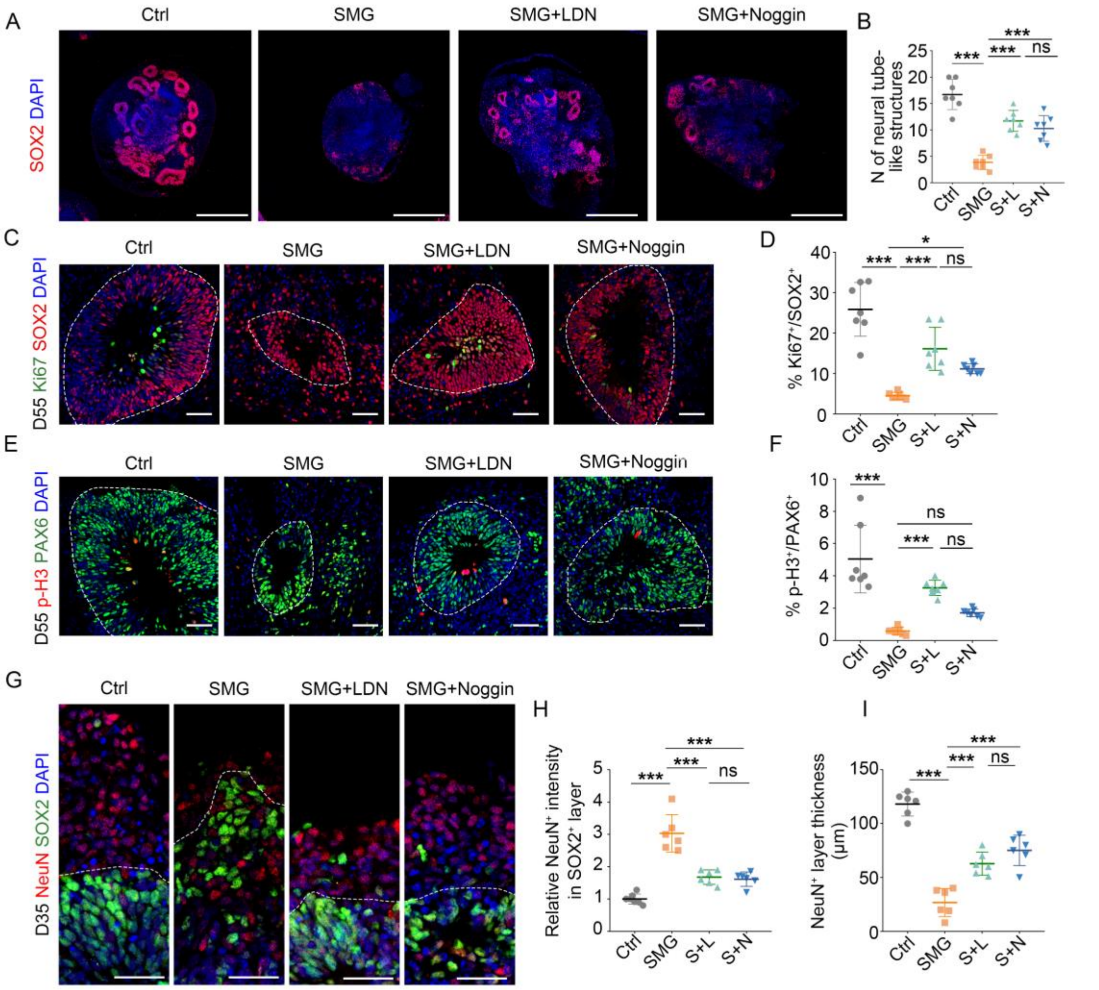


**Figure S2. Inhibition of the BMP signaling significantly ameliorates SMG-mediated defects.** (A, B) Immunostaining and quantification of the number of neural tube-like structures per 20 μm-thick organoid section. *n* = 7 individual organoids. (C–F) Immunostaining and quantification of the cell proportion of $Ki67^+$ and $p\text{-}H3^+$ proliferating cells in $SOX2^+$ or $PAX6^+$ NSPCs in control and SMG-treated organoids at day 55. *n* = 7 individual organoids. (G–I) Immunostaining and quantification of the relative $NeuN^+$ intensity in $SOX2^+$ layer, and the thickness of $NeuN^+$ layer at day 35. *n* = 6 individual organoids. Scale bar, 500 μm (A), 50 μm (C, E, G). Statistical significance was determined by one-way ANOVA with the Dunnet post hoc test. ns: $P > 0.05$, $*P < 0.05$, $***P < 0.001$.

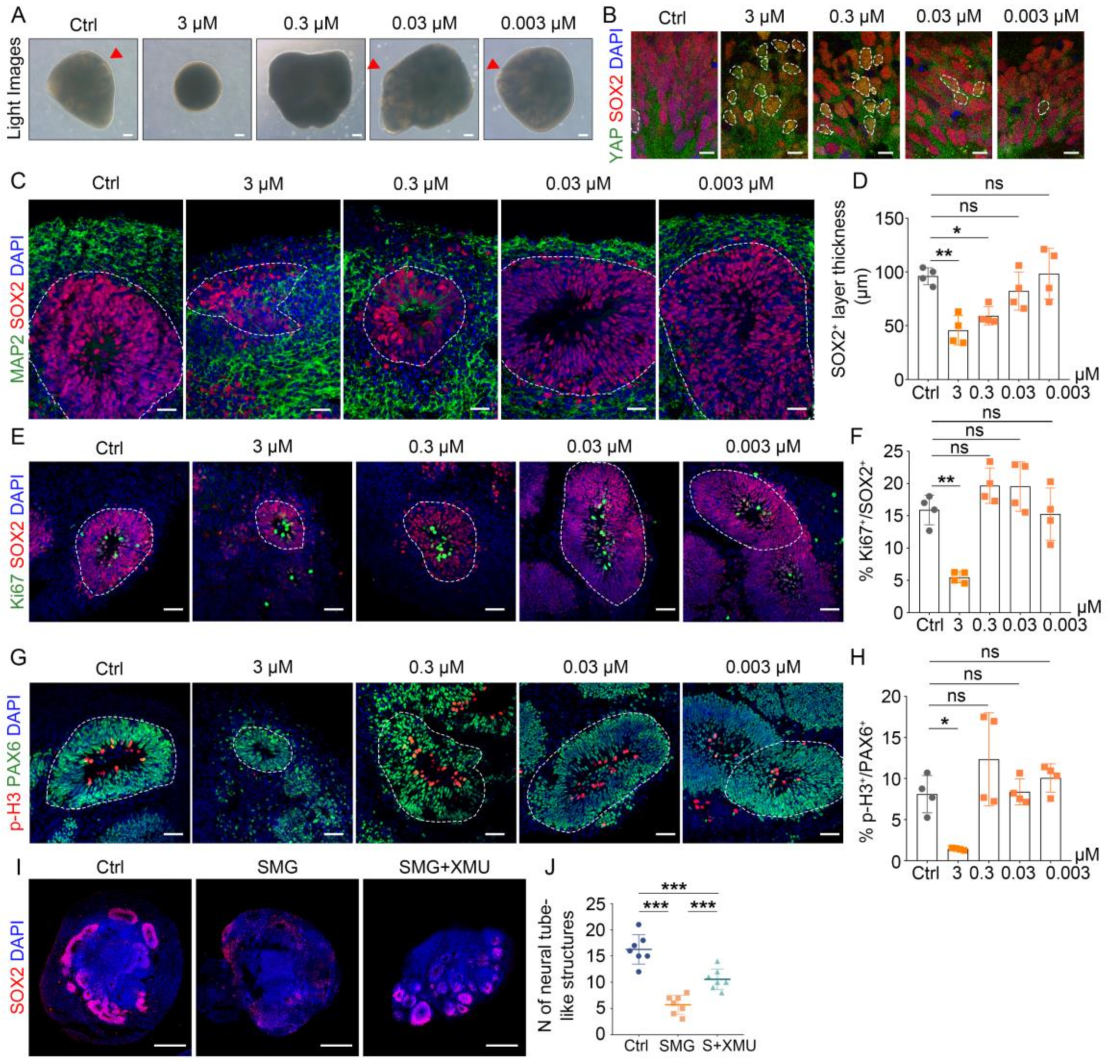


**Figure S3. YAP activation alleviates SMG-mediated defects.** (A) Light images of organoids under different dosage of XMU treatment. Red arrows indicate the apparent neural tube patterning. (B) Immunostaining of nuclear YAP in NSPCs. (C–H) Immunostaining and quantification of the thickness of $SOX2^{+}$ layer, and the cell proportion of $Ki67^{+}$ or $p\text{-}H3^{+}$ proliferating cells in $SOX2^{+}$ or $PAX6^{+}$ NSPCs. $n$ = 4 individual organoids. (I, J) Immunostaining and quantification of the number of neural tube-like structures per 20 µm-thick organoid section. Scale bar, 500 µm (I), 200 µm (A), 50 µm (C, E, G), 10 µm (B). Statistical significance was determined by one-way ANOVA with the Dunnet post hoc test. ns: $P > 0.05$, $^{*}P < 0.05$, $^{**}P < 0.01$, $^{***}P < 0.001$.

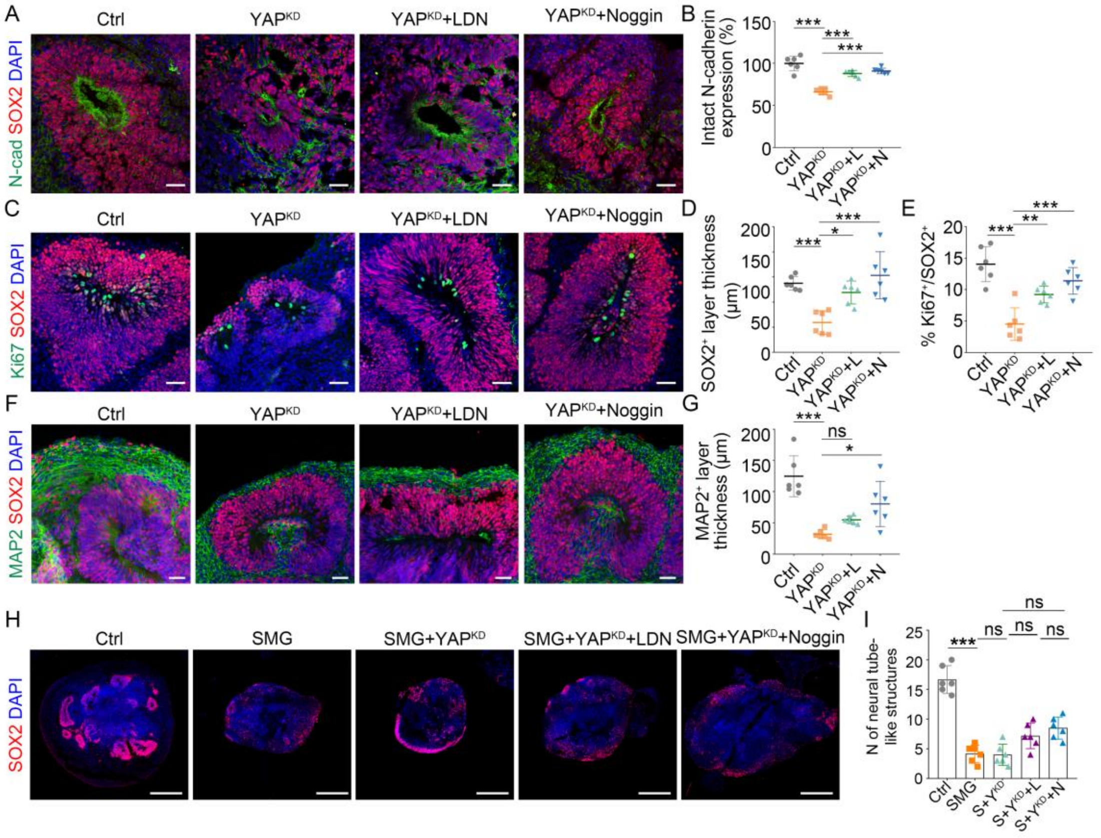


**Figure S4. Inhibition of the BMP signaling ameliorates YAP knockdown-induced defects.** (A, B) Immunostaining and quantification of the expression of N-cadherin in control, YAP-knockdown ($YAP^{KD}$), YAP-knockdown with LDN193189 ($YAP^{KD}$+LDN), and YAP-knockdown with Noggin ($YAP^{KD}$+Noggin) organoids under normal gravity conditions at day 35. $n$ = 6 individual organoids. (C–E) Immunostaining and quantification of the thickness of $SOX2^+$ layer, and the cell proportion of $Ki67^+$ proliferating cells in $SOX2^+$ NSPCs. $n$ = 6 individual organoids. (F, G) Immunostaining and quantification of the thickness of $MAP2^+$ layer. $n$ = 6 individual organoids. (H, I) Immunostaining and quantification of the number of neural tube-like structures per 20 μm-thick organoid section. $n$ = 6 individual organoids. Scale bar, 500 μm (H), 50 μm (A, C, F). Statistical significance was determined by one-way ANOVA with the Dunnet post hoc test. ns: $P > 0.05$, $*P < 0.05$, $**P < 0.01$, $***P < 0.001$.

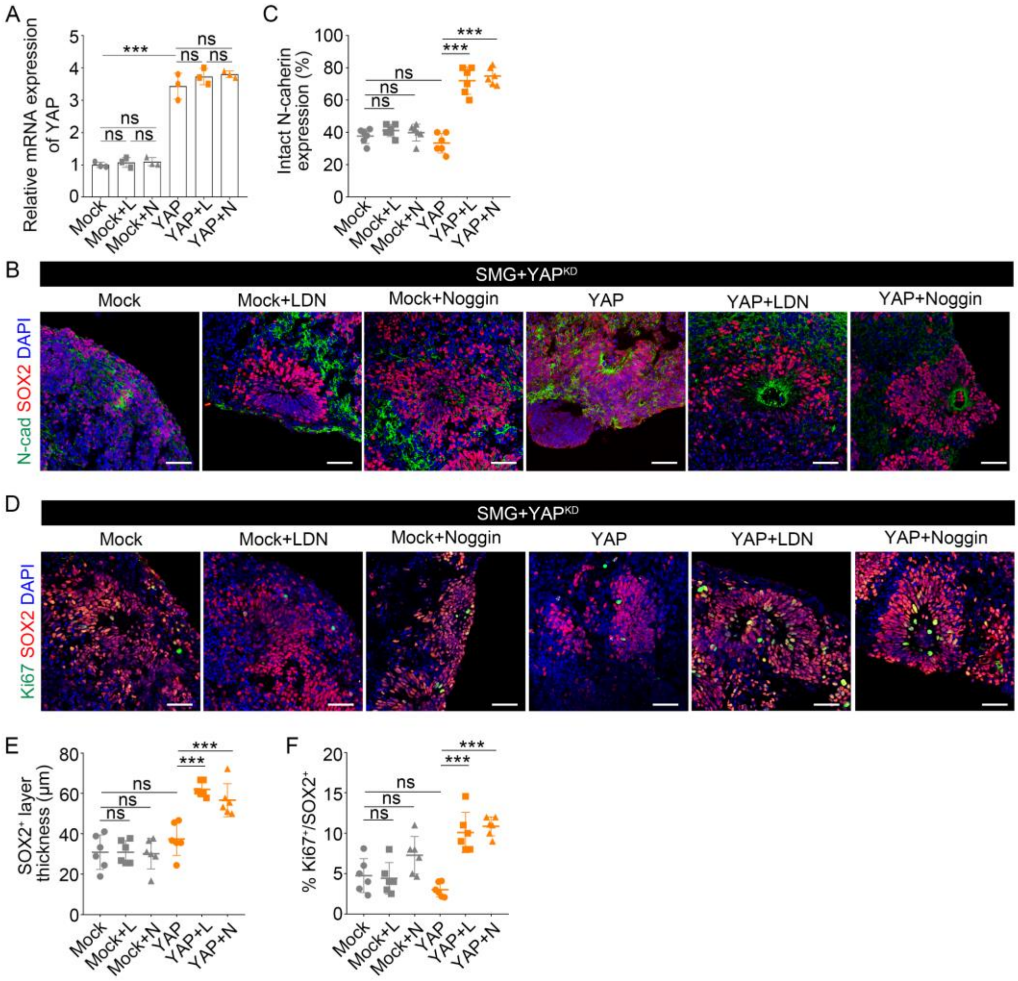


**Figure S5. Loss of YAP results in the inability of BMP inhibitors.** (A) qRT-PCR analysis of YAP mRNA level in YAP knockdown organoids under SMG conditions transfected with control lentivirus (MOCK), control lentivirus with LDN193189 (MOCK+LDN), control lentivirus with Noggin (MOCK+Noggin), lentiviral vector overexpressing YAP (YAP), lentiviral vector overexpressing YAP with LDN193189 (YAP+LDN), lentiviral vector overexpressing YAP with Noggin (YAP+Noggin) at day 35, demonstrating the re-expression of YAP. $n$ = 3 independent experiments. (B, C) Immunostaining and quantification of the expression of N-cadherin in YAP knockdown organoids under different groups at day 35. $n$ = 6 individual organoids. (D–F) Immunostaining and quantification of the thickness of $SOX2^+$ layer, and the cell proportion of $Ki67^+$ proliferating cells in $SOX2^+$ NSPCs. $n$ = 6 individual organoids. Scale bar, 50 μm. Statistical significance was determined by one-way ANOVA with the Dunnet post hoc test. ns: $P > 0.05$, $***P < 0.001$.

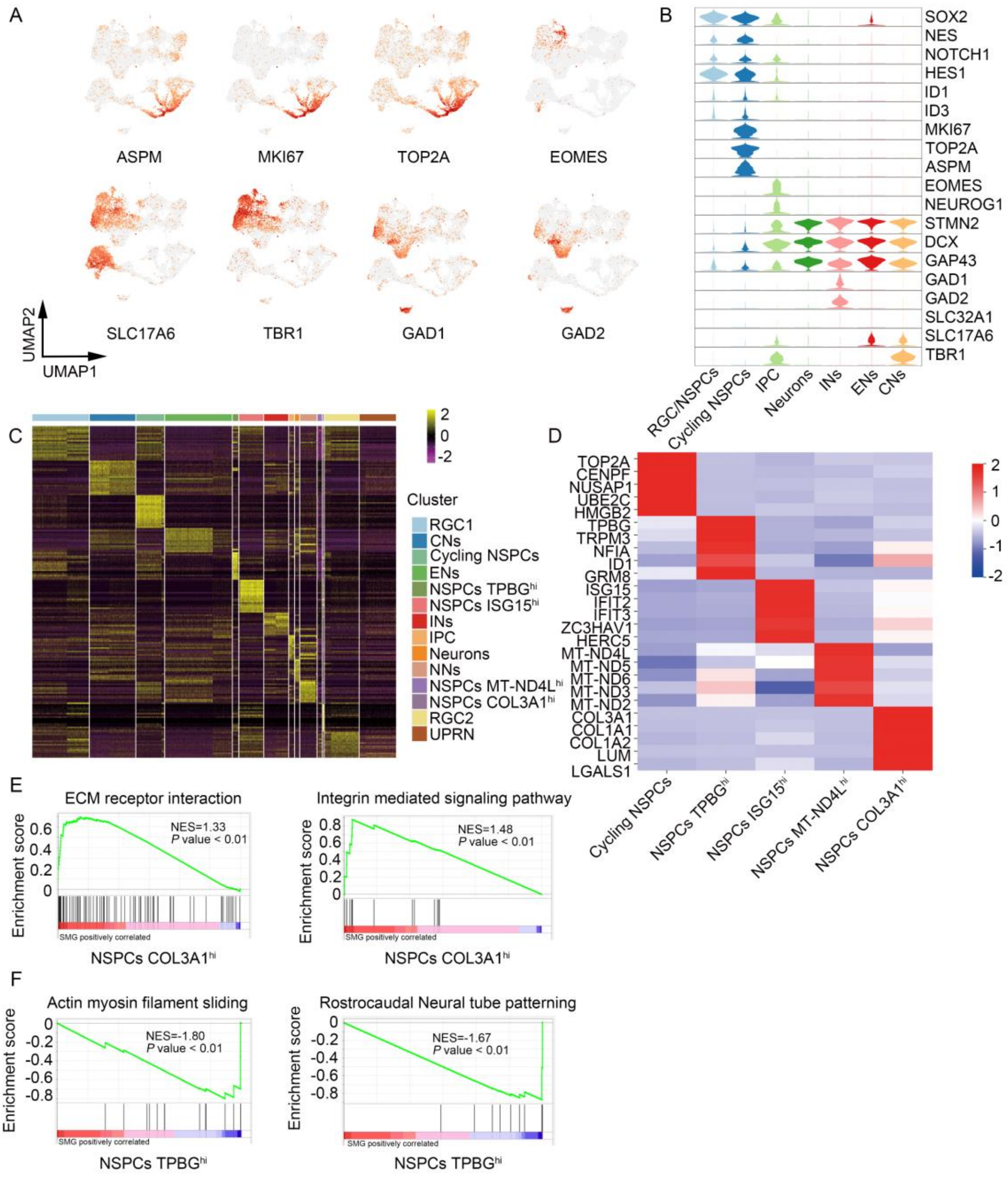


**Figure S6. Single-cell RNA-Seq reveals dysregulated NSPCs clusters in SMG-treated organoids.** (A) UMAP visualization of cell-type-specific gene expression markers in the cortical organoids. Cycling: *MKI67*, *TOP2A*, *ASPM*; intermediate progenitor cell: *EOMES*; excitatory neurons: *SLC17A6*; cortical neurons: *TBR1*; interneurons: *GAD1*, *GAD2*. (B) Expression of selected cluster-specific marker genes used for cell type classification. Violin plots show the distribution of normalized expression in cells from organoids. (C) Heatmap showing expression of the top 20 markers for each cluster. (D) Heatmap showing expression of the top 5 markers for each NSPCs cluster. (E) Gene Set Enrichment Analysis (GSEA) shows a depletion of actin myosin filament sliding and neural tube patterning genes in TPBG$^{hi}$ NSPCs from SMG-treated groups. (F) GSEA shows an increase of ECM receptor interaction and integrin mediated signaling pathway in COL3A1$^{hi}$ NSPCs from SMG-treated groups.

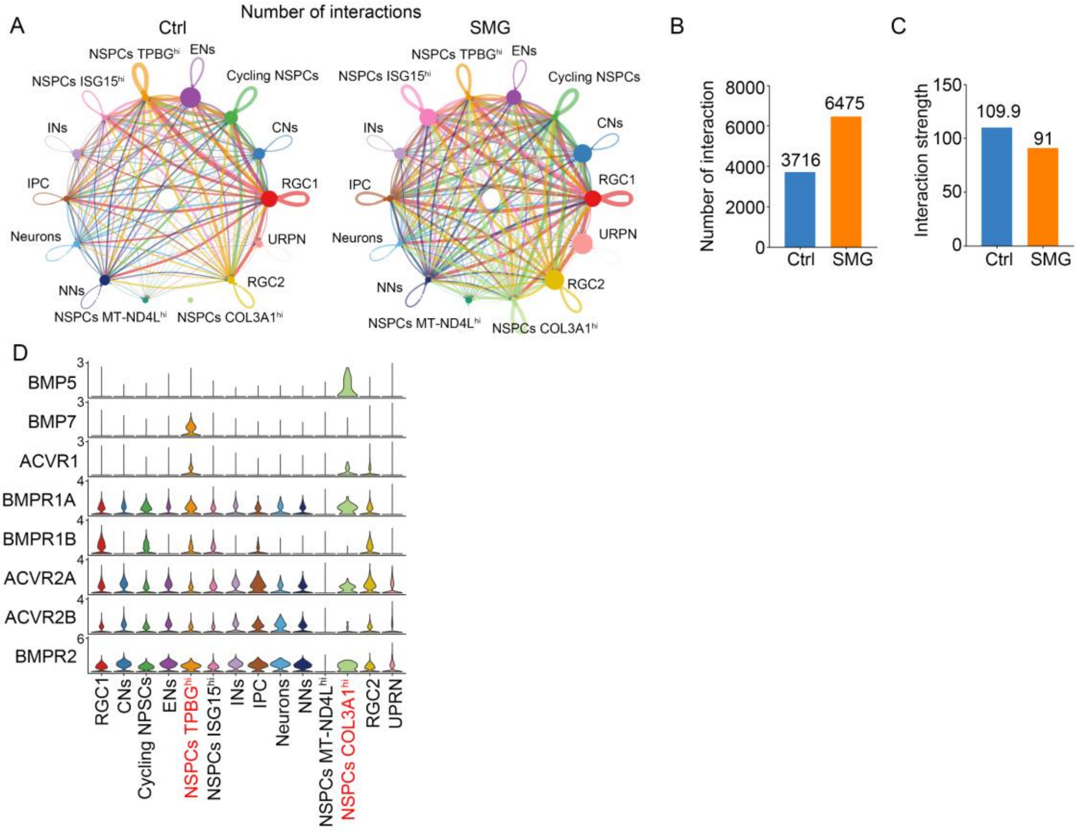


**Figure S7. Single-cell RNA-Seq reveals dysregulated NSPCs interaction in SMG-treated organoids.** (A–C) Circle plots (A) and summary data (B, C) illustrating the interaction numbers and strength of significant enriched ligand-receptor pairs among all cluster of control and SMG-treated organoids. The thickness of the line indicates the number of enrich pairs, and the arrow reflects the direction of the interaction. (D) Expression of BMP ligand and receptor genes in each cluster. Violin plots show distribution of relative expression in cells from organoids.

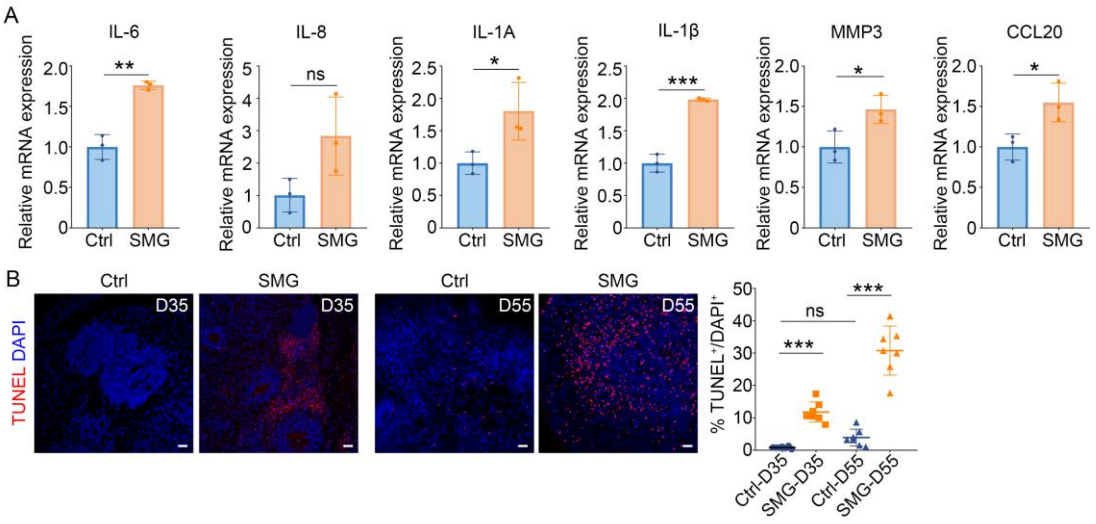


**Figure S8. SMG leads to an increase in inflammatory responses and apoptosis.** (A) The relative mRNA expression level of inflammatory responses-related gene (*IL-6*, *IL-8*, *IL-1A*, *IL-1β*, *MMP3*, *CCL20*) in SMG-treated organoids on day 35. $n = 3$ independent experiments. (B) Fluorescence images and quantification of $TUNEL^+$ cell proportion among $DAPI^+$ cells in control and SMG-treated organoids on day 35 and day 55. $n = 7$ individual organoids. Values were mean ± SD. Scale bar, 50 μm. Statistical significance was determined by unpaired, two-tailed Student's t-test (A). Statistical significance was determined by one-way ANOVA with the Dunnet post hoc test (B). ns: $P > 0.05$, $*P < 0.05$, $**P < 0.01$, $***P < 0.001$.

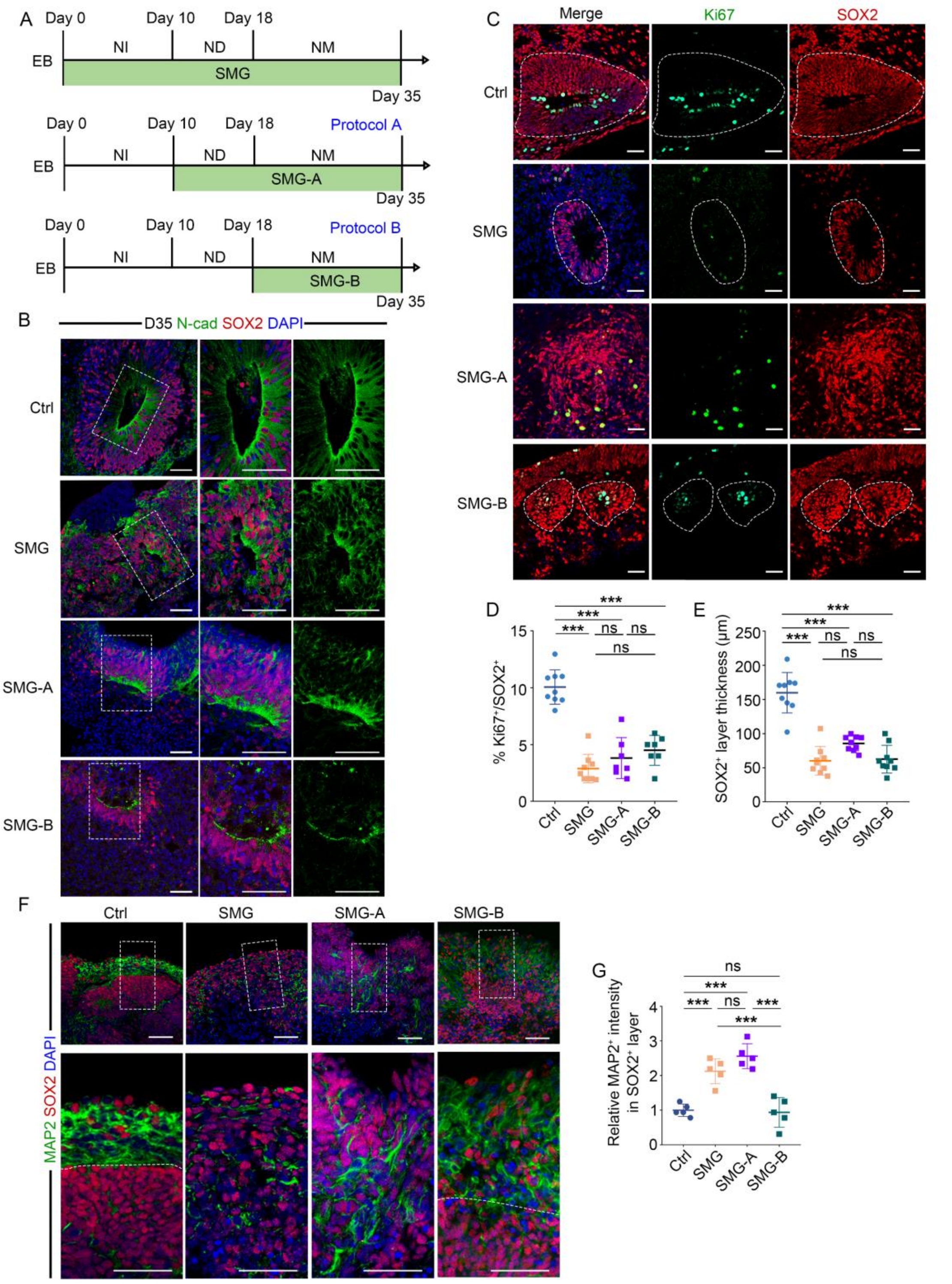


**Figure S9. SMG intervention at different time points leads to neurologic damage.** (A) Schematic of experimental design. NI, neural induction; ND, neural differentiation; NM, neural maturation. In protocol A, the SMG treatment started on the ND stage, while the SMG exposure started on NM stage in protocol B. (B) Immunostaining of the expression of N-cadherin. (C–E) Immunostaining and quantification of the thickness of $SOX2^+$ layer, and the cell proportion of $Ki67^+$ proliferating cells in $SOX2^+$ NSPCs. *n* = 9 individual organoids. (F, G)

Immunostaining and quantification of the proportion of MAP2$^+$ neurons in SOX2$^+$ layer of organoids. $n$ = 5 individual organoids. Scale bar, 50 μm. Statistical significance was determined by one-way ANOVA with the Dunnet post hoc test. ns: $P > 0.05$, ***$P < 0.001$.

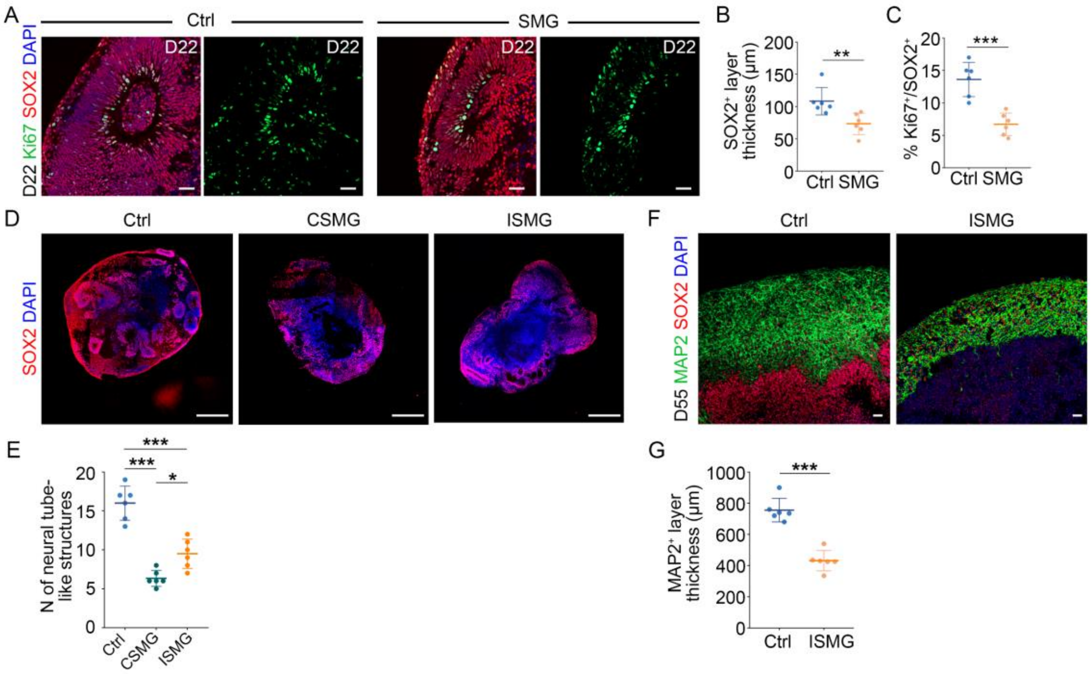


**Figure S10. SMG intervention leads to persistent neurologic damage.** (A–C) Immunostaining and quantification of the thickness of SOX2$^+$ layer, and the cell proportion of Ki67$^+$ proliferating cells in SOX2$^+$ NSPCs on day 22. The SMG intervention started on day 18, and the organoids were fixed on day 22. $n$ = 6 individual organoids. (D, E) Immunostaining and quantification of the number of neural tube-like structures per 20 μm-thick organoid section. $n$ = 6 individual organoids. (F, G) Immunostaining and quantification of the thickness of MAP2$^+$ layer on day 55. The ISMG-treated organoids were subjected to SMG stimulation on day 10 to 18 and cultured under normal gravity until day 55. $n$ = 6 individual organoids. Scale bar, 500 μm (D), 50 μm (A, F). Statistical significance was determined by unpaired, two-tailed Student's t-test (B, C, G). Statistical significance was determined by one-way ANOVA with the Dunnet post hoc test (E). *$P < 0.05$, **$P < 0.01$, ***$P < 0.001$.
